# Network toxicology-aided identification of potential toxicological mechanisms of rhodamine B in ovarian cancer: Insights from transcriptomics, molecular docking, molecular dynamics simulations, and machine learning modeling

**DOI:** 10.1016/j.jgeb.2026.100806

**Published:** 2026-08-18

**Authors:** Susen Sarker, Sheikh Abdullah Al Ashik

**Affiliations:** a Department of Biochemistry and Molecular Biology, Jagannath University, Dhaka 1100, Bangladesh; b Department of Biotechnology and Genetic Engineering, Mawlana Bhashani Science and Technology University, Santosh, Tangail 1902, Bangladesh

**Keywords:** Rhodamine B, Ovarian Cancer, Network toxicology, Molecular docking, Molecular dynamics simulations, Machine learning

## Abstract

Ovarian cancer is the most lethal gynecological malignancy, accounting for disproportionate cancer-related mortality due to late-stage diagnosis and limited therapeutic options. Despite documented cytotoxicity, genotoxicity, hepatotoxicity, and reproductive toxicity of Rhodamine B, its toxic molecular interaction landscape in ovarian cancer remains uncharacterized. Therefore, this study employed an integrative network toxicology approach, along with machine learning, molecular docking, and molecular dynamics (MD) simulations, to systematically map Rhodamine B's interactions against ovarian cancer-associated targets. Multi-database target prediction cross-referenced against curated ovarian cancer genes and differentially expressed genes from GEO datasets GSE4122 and GSE203024 of ovarian cancer identified 26 convergent targets from 331 unique predictions. Network topology (degree, betweenness, closeness, MCC, and MNC) analysis identified BCL2, BCL2L1, MMP2, and MMP9 as core targets spanning apoptosis regulation and matrix remodeling pathways. Molecular docking revealed energetically favorable binding affinities ranging from −6.4 kcal/mol (BCL2L1) to −7.8 kcal/mol (MMP2). Furthermore, MD simulations suggested stable structural behavior of the BCL2, BCL2L1, and MMP9 complexes throughout the 100 ns simulation (RMSD <3.0 Å). Post-dynamics MMPBSA calculations identified BCL2, MMP9, and BCL2L1 as the most thermodynamically stable interaction partners (ΔG = −32.593 ± 4.721, −23.439 ± 4.742, and − 21.909 ± 5.791 kcal/mol). Finally, the top machine learning models predicted the pIC_50_ value of Rhodamine B to be 4.520, denoting Rhodamine B as inactive (active threshold: 5.0) against the Caov-3 ovarian cancer cell line. These findings suggest Rhodamine B as a multi-target toxic agent, potentially disrupting apoptotic and invasive signaling associated with ovarian cancer, consistent with machine learning predictions indicating inactivity against the Caov-3 ovarian cancer cell line. Overall, these findings provide a foundation for further experimental studies to validate the computationally predicted toxic interaction profiles of Rhodamine B through cell viability, oxidative stress, apoptosis, and genotoxicity assays in ovarian cancer cell lines, as well as *in vivo* animal models.

## Highlights

• Network toxicology mapped 26 Rhodamine B targets dysregulated in ovarian cancer.
• BCL2, BCL2L1, MMP2, MMP9 identified as core toxic hubs.
• Molecular docking showed considerable binding affinities (−6.4 to −7.8 kcal/mol).
• MD simulations of 100 ns period suggested stable complexes (RMSD <3.0 Å).
• ML predicted pIC_50_ value of Rhodamine B as inactive (< 5.0) in the cell line.

## Graphical abstract

**Figure:**
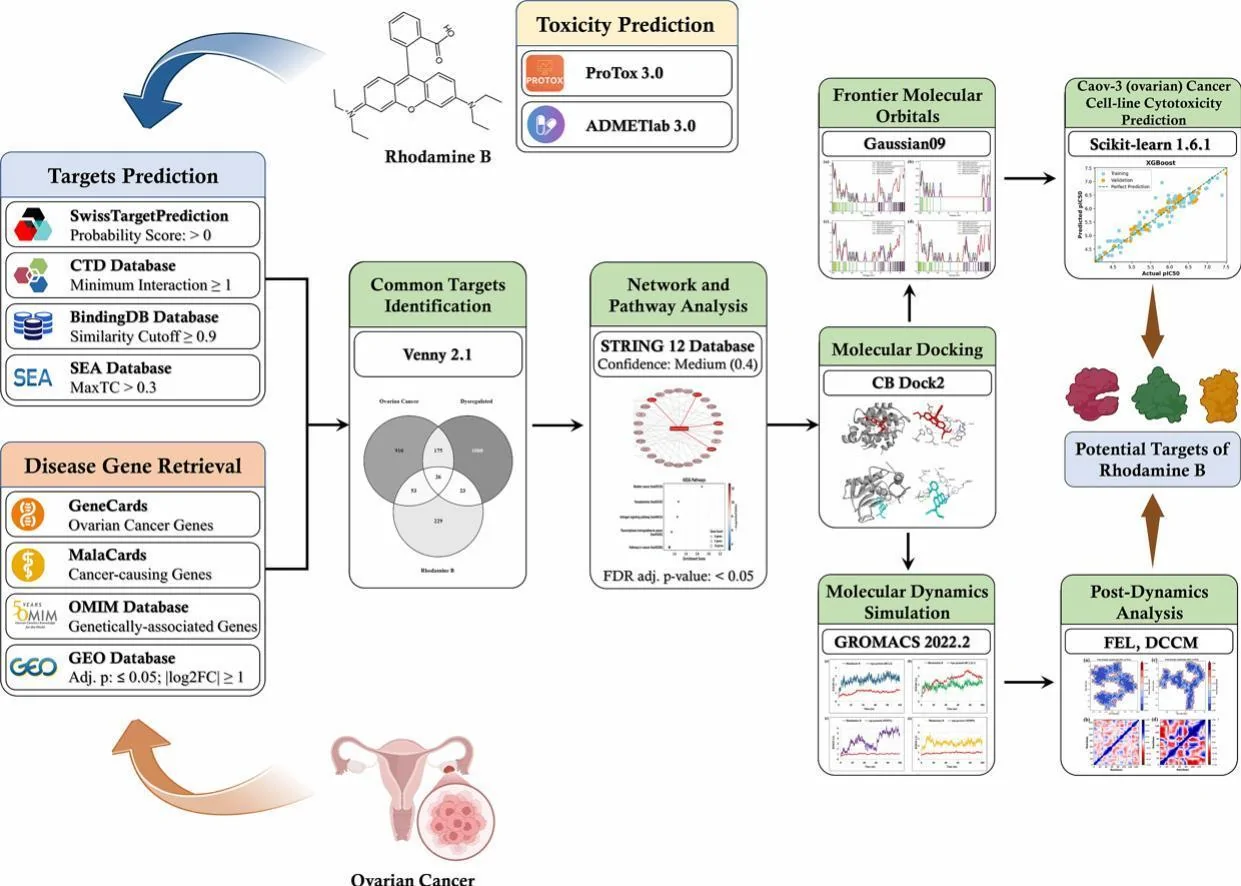

## 1 Introduction

Ovarian cancer represents one of the most aggressive malignancies of the female reproductive tract and a silent killer, characterized by complex molecular and genetic alterations that confer resistance to conventional therapies and frequently result in late-stage diagnosis.^1^ With five-year survival rates ranging from only 13–27%, this high mortality underscores the critical need for improved diagnostic methods, prognostic markers, and targeted therapeutic interventions.^2^, ^3^ At diagnosis, approximately 95% of patients present with nonspecific symptoms, including abdominal pain, bloating, and urinary urgency or frequency, while about 80% already have advanced-stage disease (III-IV), characterized by extrapelvic spread, ascites, and abdominal masses.^4^

The synthetic xanthene-based cationic red or purple color dye Rhodamine B poses significant ecological and health risks due to its persistence in aquatic environments and resistance to natural degradation processes.^5^ First synthesized in the late 19th century, it has since found widespread application in the production of textile dyes and cosmetic formulations, and is also extensively used as a fluorescent marker in biological research, as well as a tracing agent in food and pharmaceutical industries.^6^, ^7^

Its lipophilic cationic structure facilitates preferential accumulation within the mitochondrial matrix, where it disrupts BCL2-mediated outer mitochondrial membrane integrity and inhibits complex I of the electron transport chain, thereby triggering the intrinsic apoptotic cascade.^8^, ^9^, ^10^ The ingestion of Rhodamine B through food consumption may cause oxidative damage to ovarian follicles and lead to a reduction in primary, secondary, and de Graaf follicular counts.^11^, ^12^ Different transcription factors sensitive to redox alterations are triggered according to ROS concentrations and collectively manage cellular biological reactions.^12^

Besides these, Rhodamine B is widely utilized in industrial applications due to its cost-effectiveness and stable coloration properties. Despite regulatory prohibitions, Rhodamine B continues to be illegally incorporated into food products such as candies, fruit juice drinks, and hot sauce.^13^, ^14^, ^15^ Studies also suggest potential interactions of Rhodamine B with biological systems, raising concerns regarding its safety.^12^ Research has shown that Rhodamine B causes oxidative stress at the cellular level and demonstrates toxic effects on neurological, reproductive, and developmental systems.^13^, ^16^ Animal studies have also documented its carcinogenic potential.^13^ The International Agency for Research on Cancer (IARC) has designated Rhodamine B as a Group 2B carcinogen.^17^ Moreover, the California Office of Environmental Health Hazard Assessment (OEHHA) lists Rhodamine B (D&C Red No. 19) as a carcinogen, with the FDA also noting its potential to cause cancer or reproductive harm (https://oehha.ca.gov/proposition-65/chemicals/dc-red-no-19). Given these concerns, its use in food production has been explicitly banned in numerous countries and regions, including the European Union and China.^18^ Rhodamine B dye is also prohibited for food use and classified as a hazardous substance under Indonesian Health Minister Regulation No. 722/Menkes/Per/IX/1988 and Directorate General of POM Decree No. 239/Menkes/Per/V/1985, which declare certain dyes, including Rhodamine B, as dangerous materials banned for food applications.^19^

Strikingly, Rhodamine B is also recognized as a hazardous environmental pollutant and carcinogenic dye throughout Africa and South Asia, prompting the development of various remediation strategies, including adsorption, UV irradiation, chemical etching, and liquid exfoliation, aimed at eliminating this contaminant from aquatic environments.^20^, ^21^, ^22^, ^23^, ^24^, ^25^, ^26^ However, despite this established toxicological profile, cases of illegal addition of Rhodamine B are usual in food such as cotton candy, sweets, tomato sauces, chili powder, shrimp paste, and red pepper products in African and South Asian countries.^27^, ^28^, ^29^, ^30^ It is still a common practice in Southeast Asian countries, India, Bangladesh, and Pakistan, where the toxicity of Rhodamine B is often unreported and ignored due to a lack of awareness of the health complications of Rhodamine B.^31^, ^32^ The utilization of Rhodamine B in food sources contributes to water pollution and, most significantly, poses risks to human reproductive health, making it a global concern, especially for developing and underdeveloped countries, most devastatingly African, South Asian, and the South American country Argentina.^20^, ^31^, ^33^, ^34^

Despite well-documented evidence of Rhodamine B's cytotoxic, genotoxic, and reproductive toxic potential, its precise molecular targets and underlying interaction mechanisms at the proteomic level remain largely unexplored. This represents a critical research gap, as no study to date has systematically characterized the toxic molecular footprint of Rhodamine B within the specific context of ovarian cancer biology. Prior investigations have been largely restricted to Rhodamine B interaction profiles in model organisms, interaction with human serum albumin, and limited pathway-based analyses,^12^, ^35^, ^36^ leaving the system-level organization of Rhodamine B's toxic interactions unresolved.

**Fig. 1:**
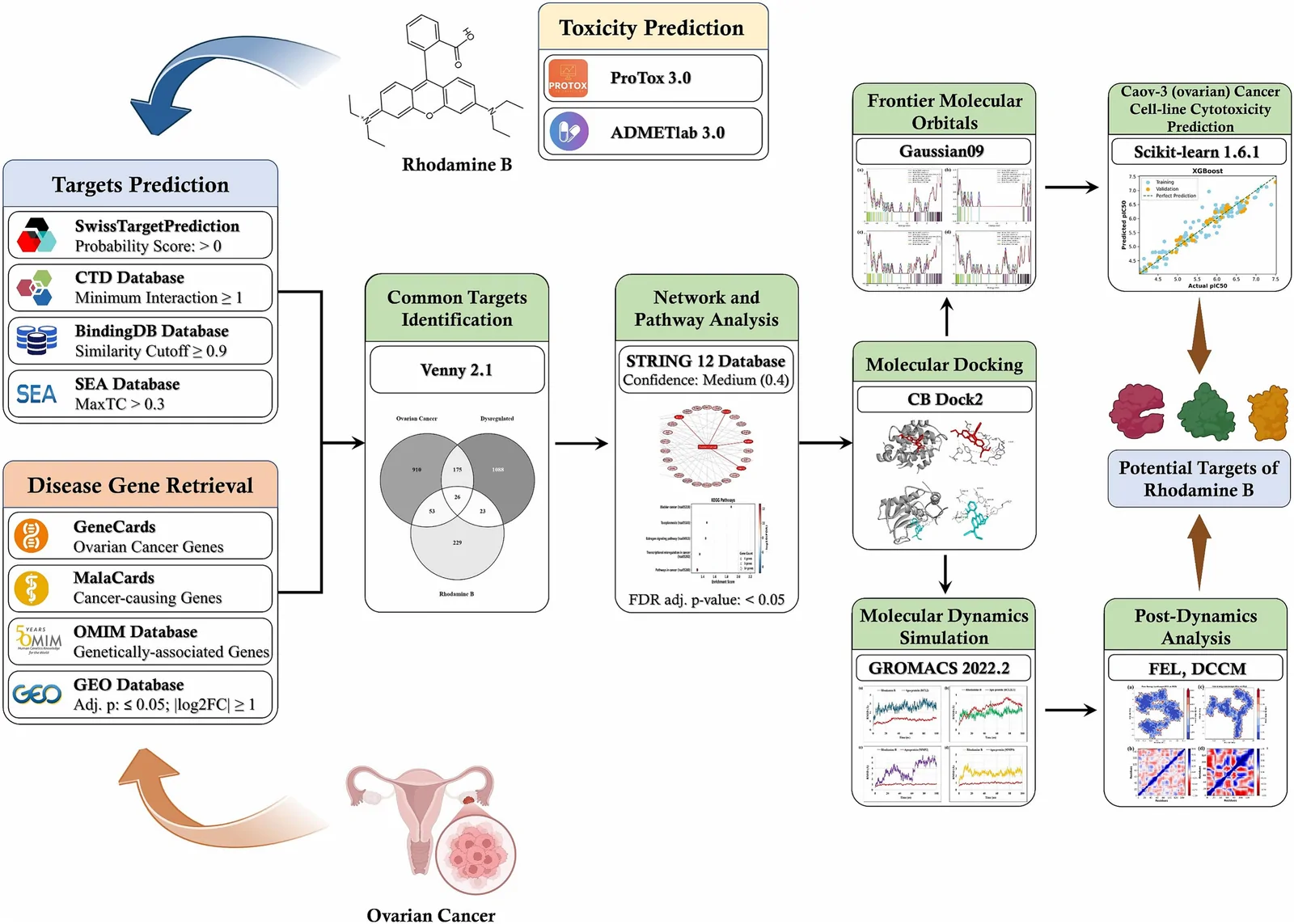
Flow diagram of the study presenting Rhodamine B's activity against ovarian cancer in modulating potential targets.

Therefore, distinct from previous network toxicology studies of environmental toxicants, which have largely relied on target prediction, network analysis, molecular docking, and MD simulations, the present study aimed to integrates network toxicology with transcriptomic cross-validation from independent GEO datasets, molecular docking, MD simulations, frontier molecular orbital (FMO) theory and machine learning-based bioactivity prediction, within a single, unified framework to systematically characterize the toxic molecular interaction landscape of Rhodamine B in ovarian cancer. The flow diagram of the study is presented in Fig. 1.

## 2 Methods

### 2.1 *In silico* organ and end-point toxicity analysis

To evaluate the organ-level and endpoint-specific toxicity profile of Rhodamine B, *in silico* predictions were performed using ProTox 3.0 (https://tox.charite.de/protox3/) and ADMETlab 3.0 (https://admetlab3.scbdd.com/).^37^, ^38^ ProTox 3.0 is a machine-learning-based web server that predicts organ toxicity and key toxicological endpoints, including mutagenicity, carcinogenicity, BBB penetration, and ecotoxicity, reporting each as a probability score with an active or inactive classification. On the other hand, ADMETlab 3.0 is a deep learning-integrated ADMET prediction platform covering an extensive toxicity panel, wherein compounds are classified as toxic at a probability threshold exceeding 0.70. The concurrent use of both tools was intended to enhance prediction reliability through cross-platform concordance, thereby providing a robust basis for prioritizing toxicity liabilities.

### 2.2 Prediction of potential targets

To identify potential molecular targets for compounds of interest in *Homo sapiens*, a comprehensive computational target-prediction approach was employed using multiple complementary databases and prediction platforms. SwissTargetPrediction (http://www.swisstargetprediction.ch/)^39^ was utilized as the primary reverse pharmacophore screening tool to predict macromolecular targets based on structural similarity to known bioactive compounds through a combination of 2D and 3D molecular descriptors. Targets with prediction scores greater than 0 were retained to ensure broad coverage of potential interactions. The Comparative Toxicogenomics Database (CTD; http://ctdbase.org/)^40^ was queried to identify curated chemical-gene/protein interactions derived from published literature, with a minimum threshold of at least one documented interaction to establish empirical evidence of molecular engagement. BindingDB (https://www.bindingdb.org/),^41^ a public repository of experimentally measured binding affinities between small molecules and proteins, was interrogated to identify structurally similar ligands with known target profiles, applying a stringent similarity cutoff of at least 0.9 to ensure high structural concordance and predictive reliability. Additionally, the Similarity Ensemble Approach (SEA; http://sea.bkslab.org/)^42^ was implemented to compare compound chemical similarity networks against annotated ligand sets, with targets exhibiting a Maximum Tanimoto Coefficient (MaxTC) greater than 0.3 being considered significant to capture pharmacologically relevant relationships.

### 2.3 Analysis of disease-relevant targets

Disease-associated genes were retrieved from GeneCards (https://www.genecards.org/),^43^ a comprehensive database that aggregates information from multiple sources to provide detailed gene annotations, functional descriptions, and disease associations, thereby enabling robust target identification. The OMIM database (https://www.omim.org/)^44^ was queried to identify genes with documented genetic associations to human diseases, as OMIM catalogs comprehensive information on genetic disorders and their molecular basis through curated literature evidence. To complement these curated resources, the Gene Expression Omnibus (GEO) database (https://www.ncbi.nlm.nih.gov/geo/)^45^ was systematically screened to retrieve transcriptomic datasets relevant to the disease of interest, as GEO provides a public repository of high-throughput gene expression data that enables identification of differentially expressed genes across disease and control conditions. Differential expression analysis was performed with stringent statistical thresholds applied (adjusted *p*-value <0.05 and |log₂ fold change| ≥ 1) to identify genes exhibiting significant expression alterations to ensure the selection of biologically meaningful targets while minimizing false-positive discoveries. Additionally, the MalaCards database (https://www.malacards.org/)^46^ was interrogated to obtain integrated disease-centric information, as MalaCards consolidates data from diverse sources to provide comprehensive disease annotations, associated genes, and pathways. The integration of these complementary databases was designed to cross-validate target candidates through multiple lines of evidence: genetic association, expression dysregulation, and disease annotation, thereby generating a high-confidence list of disease-relevant molecular targets.

### 2.4 Identification of common molecular targets

To elucidate shared molecular targets between rhodamine B exposure and ovarian cancer pathogenesis, Venny 2.1 (https://bioinfogp.cnb.csic.es/tools/venny/)^47^ was selected due to its robust capability to visualize overlapping elements between multiple datasets. Target gene sets associated with rhodamine B and ovarian cancer were compiled to identify common targets present in both datasets, with results visualized through a Venn diagram depicting unique and shared elements.

### 2.5 Protein-protein interaction network analysis

To identify and characterize potential protein-protein interactions (PPIs) and functional relationships, a protein interaction network was constructed.^48^ The interaction network was generated using the STRING 12 database (https://string-db.org/),^49^ which integrates multiple sources of protein interaction data, including experimental evidence, computational prediction methods, and public literature mining. The network interaction setting was maintained with a minimum required interaction score threshold of 0.4 (medium confidence), which represents a balanced approach between sensitivity and specificity in detecting biologically relevant interactions while filtering out potentially spurious connections. Based on PPIs, the gene ontology and pathways enrichments were analyzed by computing biological process (BP), molecular function (MF), KEGG pathways, and Reactome pathways.

### 2.6 Identification and analysis of hub proteins

To identify critical hub proteins within the PPI network, network topology analysis was conducted using Cytoscape 3.10.4.^50^ The constructed PPI network was imported into Cytoscape 3.10.4, where network visualization and initial quality assessment were performed to ensure proper node and edge representation. Subsequently, the cytoHubba app^2^ from Cytoscape was applied to calculate multiple topological scoring metrics for each protein node, enabling systematic ranking based on their connectivity and positional importance within the network architecture. The cytoHubba app was specifically utilized to rank nodes in biological networks through various topological centrality measures such as degree, betweenness, closeness, MCC, and MNC.

### 2.7 Molecular docking analysis of protein-ligand interactions

To evaluate the binding affinity and interaction patterns between identified hub proteins and Rhodamine B, molecular docking was performed.^51^ CB-Dock2 (http://cao.labshare.cn:11080/cb-dock2),^52^ was employed, which uses AutoDock VINA 1.1.2 to integrate automated cavity detection and molecular docking using an advanced algorithm. The three-dimensional structures of target hub proteins were retrieved from the RCSB PDB database (https://www.rcsb.org/) and prepared by removing water molecules, heteroatoms, and co-crystallized ligands, while ligand structures were obtained in SDF format and energy-minimized before docking. The docking protocol was validated using the respective co-crystallized ligand of target proteins by comparing their pre-docking and post-docking poses.

### 2.8 Frontier molecular orbitals analysis

To calculate pre-docking and post-docking electronic energy and reactivity properties of Rhodamine B, frontier molecular orbitals analysis was performed.^53^, ^54^ The analysis was performed using Density Functional Theory (DFT) calculations through the B3LYP algorithm and the 6-311G basis under Gauss View 06 software,^55^ utilizing equations:

(1) $$\mathrm{Gap}\;\left(\Delta E\right)=\left(E_{\text{LUMO}}-E_{\text{HOMO}}\right)$$

(2) $$\text{Hardness},\eta =\frac{\left(E_{\text{LUMO}}-E_{\text{HOMO}}\right)\;}{2}$$

(3) $$\text{Softness},S=\frac{1}{2\eta }$$

(4) $$\text{Electronegativity},\chi =-\frac{\left(E_{\text{LUMO}}+E_{\text{HOMO}}\right)\;}{2}$$

(5) $$\text{Mean Energy},\mu =\frac{\left(E_{\text{LUMO}}+E_{\text{HOMO}}\right)\;}{2}$$

(6) $$\text{Electrophilicity},\omega =\frac{\mu^{2}}{2\eta }$$

The results of frontier molecular orbitals and density of states of Rhodamine B were visualized using GaussSum Software.

### 2.9 MD simulation and binding stability assessment

MD simulation was employed to assess the structural stability and conformational dynamics between proteins-Rhodamine B complexes under physiological conditions.^56^ MD simulation was performed using GROMACS 2022.2.^57^ The protein-ligand complexes obtained from molecular docking were prepared by assigning the AMBER99SB-ILDN force field for protein parameterization and the General AMBER Force Field (GAFF) for ligand topology generation, followed by solvation in a cubic water box using the TIP3P water model with a minimum distance of 1.0 nm from the protein surface, and neutralization through addition of counter ions to achieve physiological ionic strength.^58^ Energy minimization was performed using the steepest descent algorithm to eliminate steric conflicts, followed by equilibration in two phases: NVT ensemble at 300 K for 100 ps using the V-rescale thermostat, and NPT ensemble at 300 K and 1 atm for 100 ps using the Parrinello-Rahman barostat to achieve proper density and pressure stabilization. Production MD simulations were subsequently conducted for 100 ns at 300 K and 1 atm with a time step of 2 fs, where coordinates were saved every 10 ps for trajectory analysis. Multiple structural and energetic parameters were computed to comprehensively characterize system behavior: root mean square deviation (RMSD) was calculated to evaluate overall structural stability and convergence of the protein backbone and ligand heavy atoms relative to the initial structure; root mean square fluctuation (RMSF) was determined to identify residue-wise flexibility, and intramolecular hydrogen bonds were monitored to evaluate the stability of protein secondary structure and internal interactions.

### 2.10 Post-dynamics analysis

Molecular mechanics Poisson-Boltzmann surface area (MMPBSA)^59^ calculations were performed using gmx_MMPBSA to estimate binding free energies between protein-ligand complexes.^60^ Furthermore, the dynamic cross-correlation matrix (DCCM) analysis was conducted to reveal correlated and anti-correlated motions between residue pairs, and free energy landscapes (FEL) were constructed of complexes using principal component analysis to visualize conformational space exploration and identify dominant conformational states that provide a mechanistic understanding of protein flexibility and ligand-induced conformational changes.

### 2.11 Machine learning-based cytotoxicity prediction

Machine learning provides an efficient strategy for predicting compound cytotoxicity and biological activity through data-driven modeling approaches.^54^, ^61^ To evaluate whether Rhodamine B has a cytotoxic effect against the Caov-3 ovarian cancer cell line, six supervised regression algorithms were employed using a curated dataset of 201 experimentally validated compounds after removal of duplicates and missing data points. In general, Rhodamine B is expected to be inactive against the ovarian cancer cell line, which means Rhodamine B should be unable to inhibit ovarian cancer in a cell line study. The dataset was retrieved from the ChEMBL database (https://www.ebi.ac.uk/chembl)^62^ with ID: CHEMBL614379 and partitioned into training and validation subsets at an 80:20 ratio *via* train_test_split, allocating 160 compounds for training and 41 for held-out validation. Due to the limited number of data points, Morgan features were selected and calculated using RDKit to break down features into the fingerprint level.^63^ Additionally, the SHAP (SHapley Additive exPlanations) method was applied to identify the top 30 most important features (∼7 per data point) to balance the selection of the number of features against the number of data points most effectively. Model generalizability was further assessed through five-fold cross-validation (KFold, n_splits = 5) with predictions generated using cross_val_predict. Performance was quantified using mean absolute error (MAE), root mean square error (RMSE), and coefficient of determination (R^2^) across training, validation, and cross-validation sets, with fold-wise standard deviations reported to assess model stability. The key hyperparameters of five algorithms, implemented in scikit-learn 1.6.1^64^ and XGBoost *via* XGBRegressor, were optimized as follows: Support Vector Regressor, which identifies an optimal regression hyperplane within a defined tolerance margin; K-Nearest Neighbors, a non-parametric instance-based learner averaging responses from the k nearest training samples; Decision Tree, a hierarchical rule-based model that recursively partitions the feature space; Random Forest, a variance-reducing ensemble of decorrelated trees trained *via* bootstrap aggregation; Gradient Boosting, a sequential ensemble that additively fits weak learners to residuals of prior iterations; and XGBoost, a regularized gradient-boosted tree implementation incorporating L1 and L2 penalties to control model complexity yielding the most favorable bias-variance balance for reliable and generalizable cytotoxicity prediction.

## 3 Results

### 3.1 Toxicity profile of rhodamine B from *in silico* tools and literature

The toxicity profile of Rhodamine B was delineated prior to subsequent analysis, where ProTox-3 flagged the compound as active for respiratory toxicity (0.83), mutagenicity (0.62), nephrotoxicity (0.60), and BBB penetration (0.61), while cardiotoxicity, carcinogenicity, immunotoxicity, and cytotoxicity were predicted as inactive. Furthermore, ADMETLab 3.0 (threshold >0.70) corroborated and extended these concerns, identifying genotoxicity (1.000), AMES mutagenicity (0.995), skin sensitization (1.000), respiratory toxicity (1.000), eye irritation (1.000), DILI (0.912), eye corrosion (0.996), drug-induced nephrotoxicity (0.833), and hERG blockade at 10 μM (0.851) as the principal liabilities (Table 1). Although the reproductive toxicity of Rhodamine B is supported by the literature,^11^, ^12^ the reproductive toxicity prediction profile is unavailable and considered a limitation for current *in silico* tools.

**Table 1:** Common active toxicities predicted by both ProTox-3 and ADMETLab 3.0.

Toxicity Parameter	ProTox-3 Prediction (Probability Score)	ADMETLab 3.0 Prediction (Probability Score)
Nephrotoxicity	Active (0.60)	Toxic (0.833)
Respiratory Toxicity	Active (0.83)	Toxic (1.000)
Mutagenicity or Genotoxicity	Active (0.62)	Toxic (AMES: 0.995, Genotoxicity: 1.000)

### 3.2 Targets of rhodamine B

The integrated analysis across six distinct databases yielded a total of 353 predicted targets of *Homo sapiens* for rhodamine B, which was reduced to 331 unique targets after duplicate removal. Pharmamapper contributed the largest number of predicted targets with 200 entries, followed by Swiss Target Prediction with 100 targets, and TargetPred with 36 targets. The remaining databases provided fewer but potentially more experimentally validated targets: SEA identified 5 targets, STITCH predicted 10 targets, while ChEMBL and DGIdb each contributed 1 target.

### 3.3 Ovarian cancer-associated targets

The compilation of ovarian cancer-related genes from three major disease databases yielded 1183 genes, which were reduced to 1164 unique genes after removing duplicates. MalaCards provided the most extensive gene collection, with 1161 entries; OMIM contributed 7 genes, and the KEGG Disease database identified 15 genes. Complementary transcriptomic analysis of Gene Expression Omnibus datasets identified 1400 differentially expressed genes, which were reduced to 1312 after removing duplicates. The GSE4122 dataset contributed 956 differentially expressed genes, while GSE203024 provided 434 genes.

### 3.4 Shared targets between rhodamine B and ovarian cancer

**Fig. 2:**
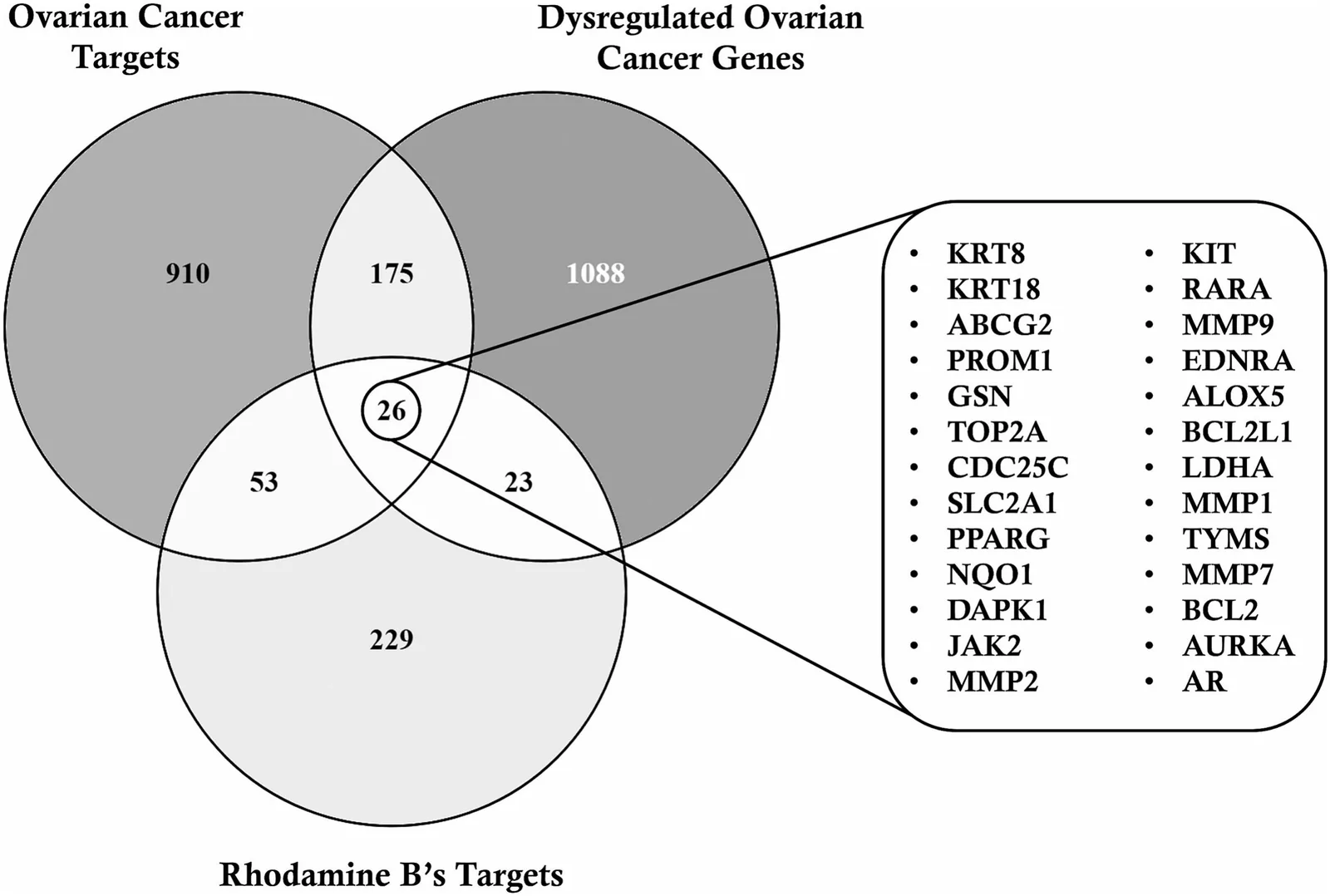
Identification of shared targets between Rhodamine B's targets and targets associated with Ovarian Cancers.

Performing target overlap analysis is necessary to identify potential intersections between Rhodamine B's molecular targets and ovarian cancer-associated proteins. The Venn diagram analysis (Fig. 2) revealed that among the total dataset comprising ovarian cancer targets, dysregulated ovarian cancer genes, and Rhodamine B's targets, there were 26 shared targets common to all three categories, representing the core intersection. Rhodamine B demonstrated interactions with 331 total targets, of which 53 targets were exclusively shared with ovarian cancer targets, 23 targets overlapped solely with dysregulated ovarian cancer genes, and 229 targets were unique to Rhodamine B without overlap with ovarian cancer-related proteins. The ovarian cancer targets encompassed 1085 proteins total, with 910 being exclusive to this category and 175 shared specifically with dysregulated ovarian cancer genes. Dysregulated ovarian cancer genes comprised 1263 total entities, with 1088 being unique to this category. The 26 core shared targets included critical proteins such as KRT8, KRT18, ABCG2, PROM1, GSN, TOP2A, CDC25C, SLC2A1, PPARG, NQO1, DAPK1, JAK2, MMP2, KIT, RARA, MMP9, EDNRA, ALOX5, BCL2L1, LDHA, MMP1, TYMS, MMP7, BCL2, AURKA, and AR, which represent diverse functional categories. All target information is available in **Tables S1, S2, and S3**.

### 3.5 Functional and pathway enrichments of shared targets

**Fig. 3:**
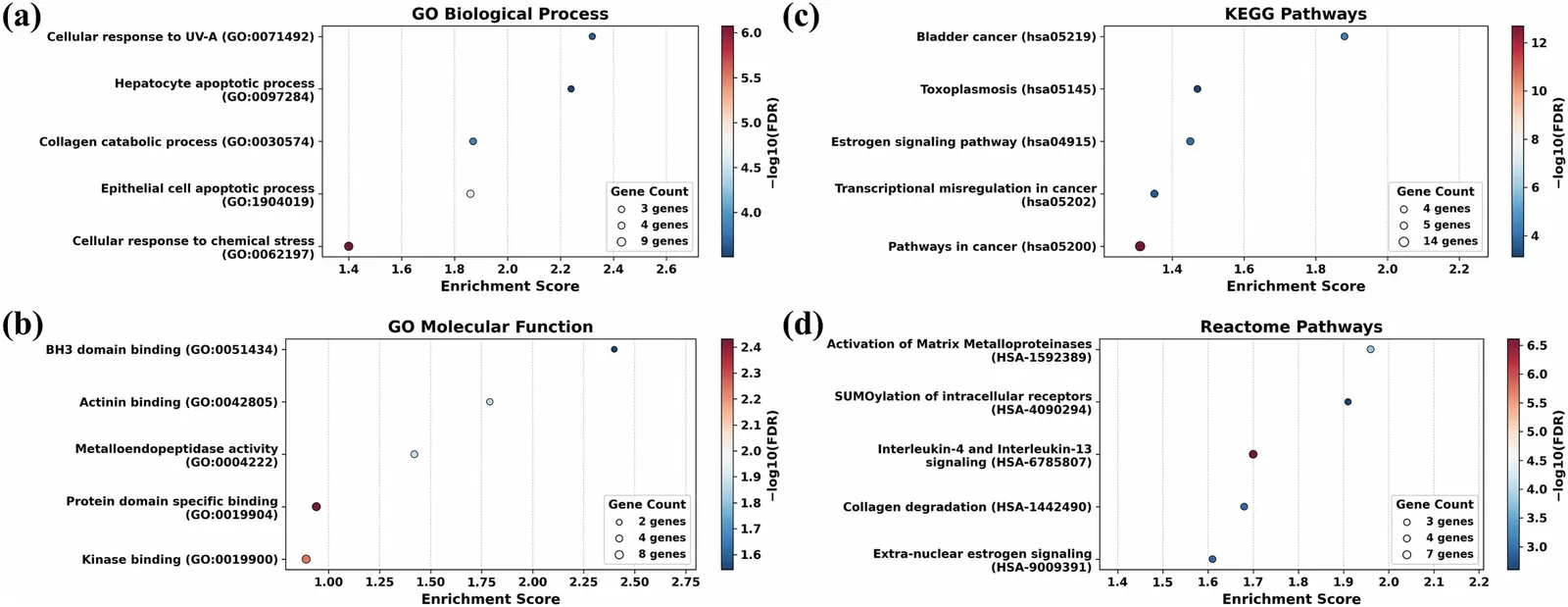
Functional and Pathway Enrichment analysis. (a) Biological Process, (b) Molecular Function, (c) KEGG pathways, and (d) Reactome pathways.

Functional enrichment analysis provides comprehensive insights into the biological relevance and mechanistic pathways underlying the shared targets between Rhodamine B and ovarian cancer. The biological process analysis revealed enrichment in cellular response to UV-A (GO:0071492), hepatocyte apoptotic process (GO:0097284), collagen catabolic process (GO:0030574), epithelial cell apoptotic process (GO:1904019), and cellular response to chemical stress (GO:0062197), with enrichment scores ranging from 1.4 to 2.6 (Fig. 3a). Molecular function analysis demonstrated enrichment in IRS1 domain binding (GO:0051434), actinin binding (GO:0042805), metalloendopeptidase activity (GO:0004222), protein domain specific binding (GO:0019904), and kinase binding (GO:0019900), with enrichment scores between 1.00 and 2.75 (Fig. 3b). KEGG pathway analysis identified key cancer-related pathways including bladder cancer (hsa05219), toxoplasmosis (hsa05145), estrogen signaling pathway (hsa04915), transcriptional misregulation in cancer (hsa05202), and pathways in cancer (hsa05200), with enrichment scores ranging from 1.4 to 2.2 (Fig. 3c). Finally, Reactome pathway analysis highlighted activation of matrix metalloproteinases (HSA-1592389), SUMOylation of intracellular receptors (HSA-4090294), interleukin-4 and interleukin-13 signaling (HSA-6785807), collagen degradation (HSA-1442490), and extra-nuclear estrogen signaling (HSA-9009391), with enrichment scores between 1.4 and 2.1 (Fig. 3d).

### 3.6 Network-based identification of core targets

**Fig. 4:**
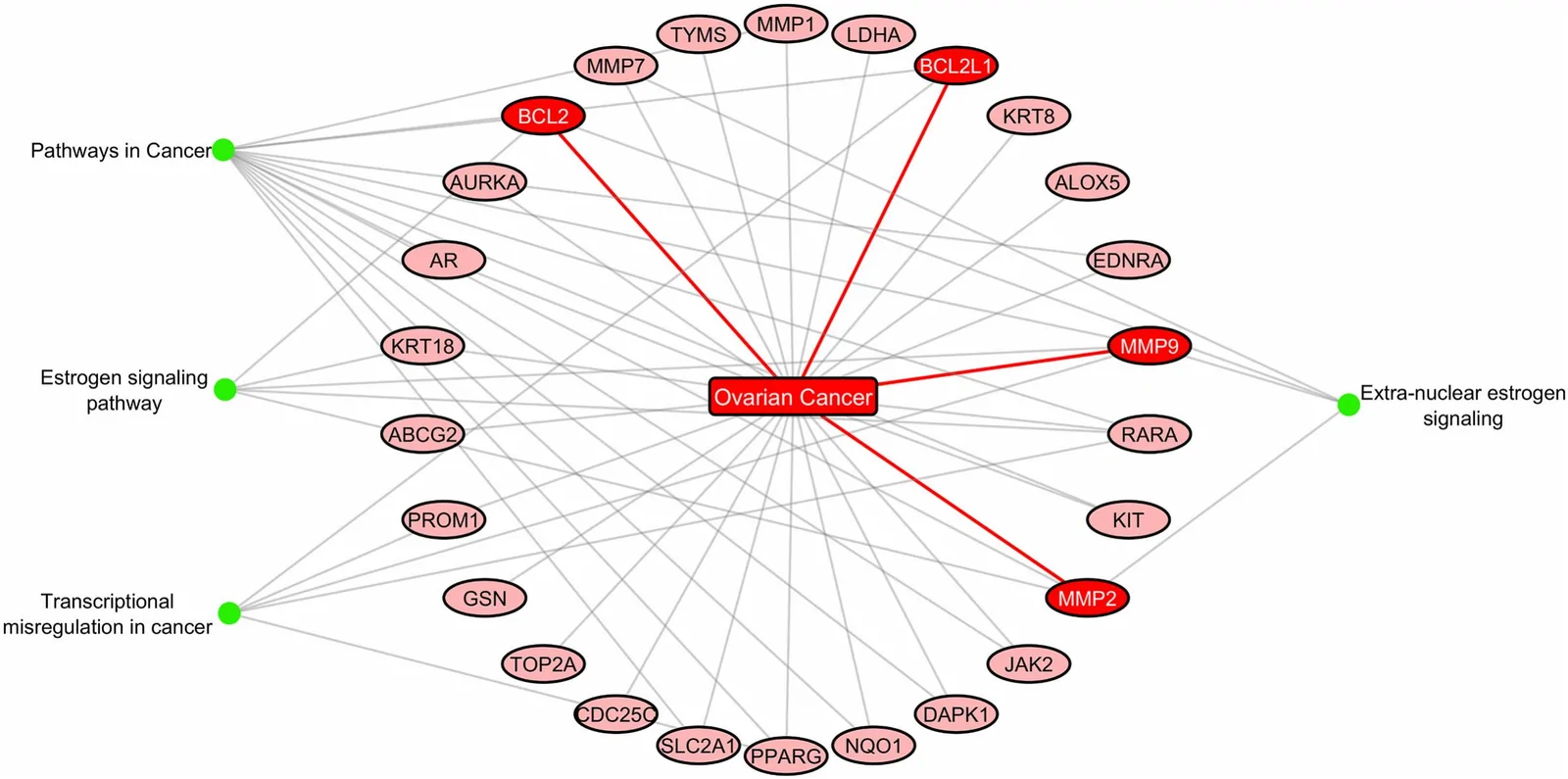
Core targets identification based on hub targets and targets associated with ovarian cancer pathways.

Network topology prioritizes the most critical targets based on multiple centrality measures. The topological analysis using degree, betweenness, closeness, MCC, and MNC parameters identified 7 hub targets: MMP9, MMP2, BCL2, BCL2L1, AR, KIT, and PPARG. Pathway-specific analysis revealed that MMP9, MMP2, and BCL2 were hub targets in the estrogen signaling pathway, while MMP9 and BCL2L1 served as hubs in transcriptional misregulation in the cancer pathway. The pathways in cancer encompassed MMP9, MMP2, BCL2, and BCL2L1 as hub targets, and the extra-nuclear estrogen signaling pathway included MMP9, MMP2, and BCL2. Cross-pathway analysis demonstrated that MMP9, MMP2, BCL2, and BCL2L1 exhibited the highest overlap across ovarian cancer-related pathways and were therefore designated as core targets (Fig. 4).

### 3.7 Molecular docking

**Fig. 5:**
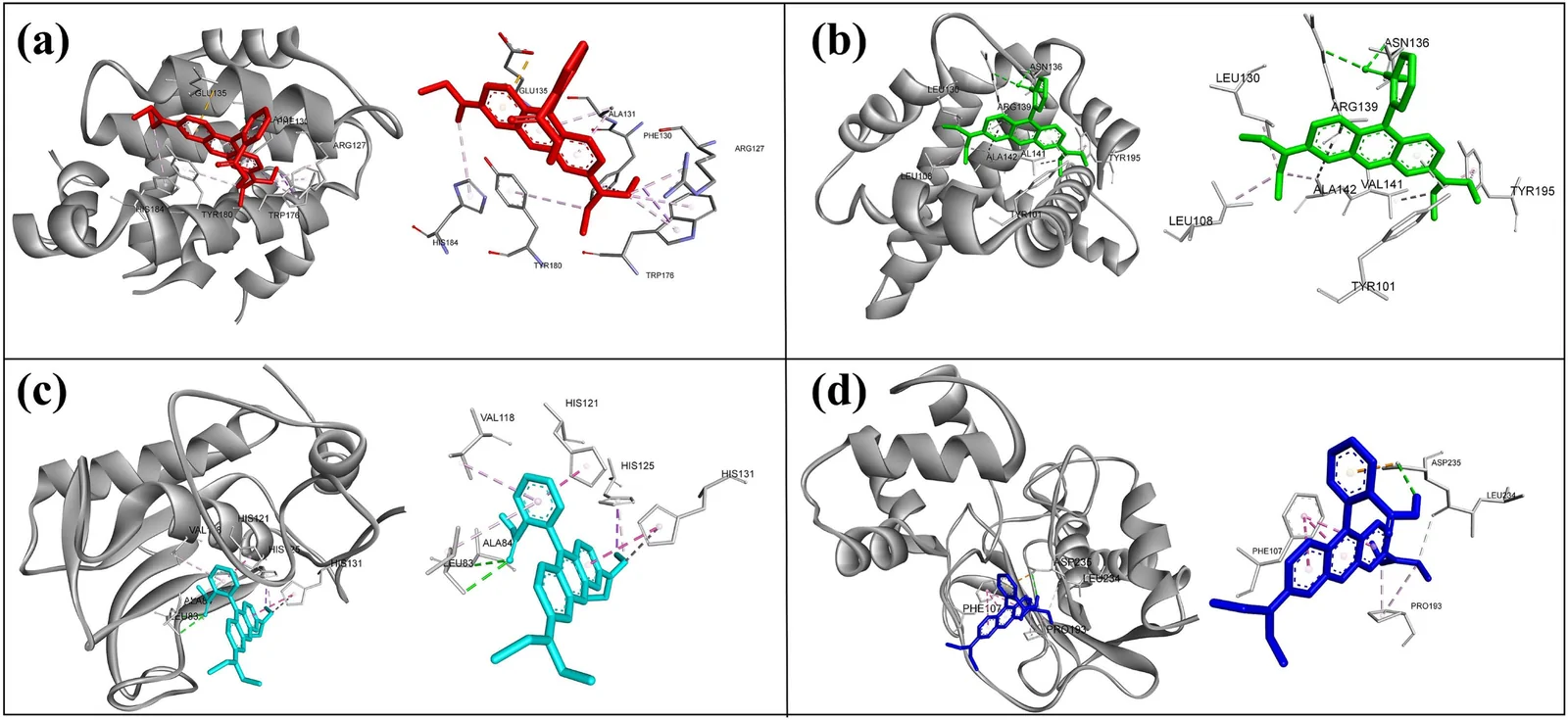
Molecular Docking Interaction Analysis. (a) BCL2, (b) BCL2L1, (c) MMP2, and (d) MMP9.

Molecular docking evaluates the binding affinity and interaction mechanisms of Rhodamine B with key cancer-related proteins BCL2, BCL2L1, MMP2, and MMP9. These targets were selected based on their high centrality in the network topology analysis, along with their involvement in the core cancer-related pathways: estrogen signaling pathway, transcriptional misregulation in the cancer pathway, pathways in cancer, and extra-nuclear estrogen signaling pathway; therefore, identifying them as the most highly connected hub targets among the 26 convergent targets. For BCL2, BCL2L1, MMP2, and MMP9, RMSDs of the pre-and post-docking poses were 0.383 Å, 1.712 Å, 0.323 Å, and 0.582 Å, respectively, which are less than the threshold of 2 Å, as visualized in (**Fig. S1**). The RMSD of less than 2 Å indicated a validated docking protocol. Further docking analysis revealed that Rhodamine B exhibited varying binding affinities across the target proteins, with MMP2 demonstrating the highest binding score of −7.8 kcal/mol, followed by MMP9 (−6.8 kcal/mol), BCL2 (−6.7 kcal/mol), and BCL2L1 (−6.4 kcal/mol) (Table 2). The structural interaction analysis showed distinct binding patterns for each protein complex (Fig. 5). BCL2 binding was primarily stabilized through hydrophobic interactions with ARG127, TRP176, PHE130, ALA131, and GLU135 within a cavity volume of 136 Å^3^, while BCL2L1 formed hydrogen bonds with TYR101, ARG139, and ASN13, complemented by hydrophobic contacts with LEU130, ALA142, and LEU108 in a larger binding pocket of 479 Å^3^. MMP2 exhibited hydrogen bonds with ALA84 and LEU83, and hydrophobic interactions with HIS131, HIS125, HIS121, and VAL118 within the largest cavity volume of 690 Å^3^. MMP9 demonstrated hydrogen bonding with LEU234 and ASP235, alongside hydrophobic interactions with LEU114, PRO193, and PHE107 in a cavity volume of 167 Å^3^.

**Table 2:** Molecular docking analysis of Rhodamine B and Protein complexes.

Proteins (PDB ID)	Docking Score (Kcal/mol)	H-bonds	Hydrophobic Interaction	Cavity Volume (Å^3^)
BCL2 (8HTS)	−6.7	N/A	ARG127, TRP176, PHE130, ALA131, GLU135	136
BCL2L1 (7Y8D)	−6.4	TYR101, ARG139, ASN13	LEU130, ALA142, LEU108	479
MMP2 (8H78)	−7.8	ALA84, LEU83	HIS131, HIS125, HIS121, VAL118	690
MMP9 (8K5Y)	−6.8	LEU234, ASP235	LEU114, PRO193, PHE107	167

### 3.8 Frontier molecular orbitals of rhodamine B

**Fig. 6:**
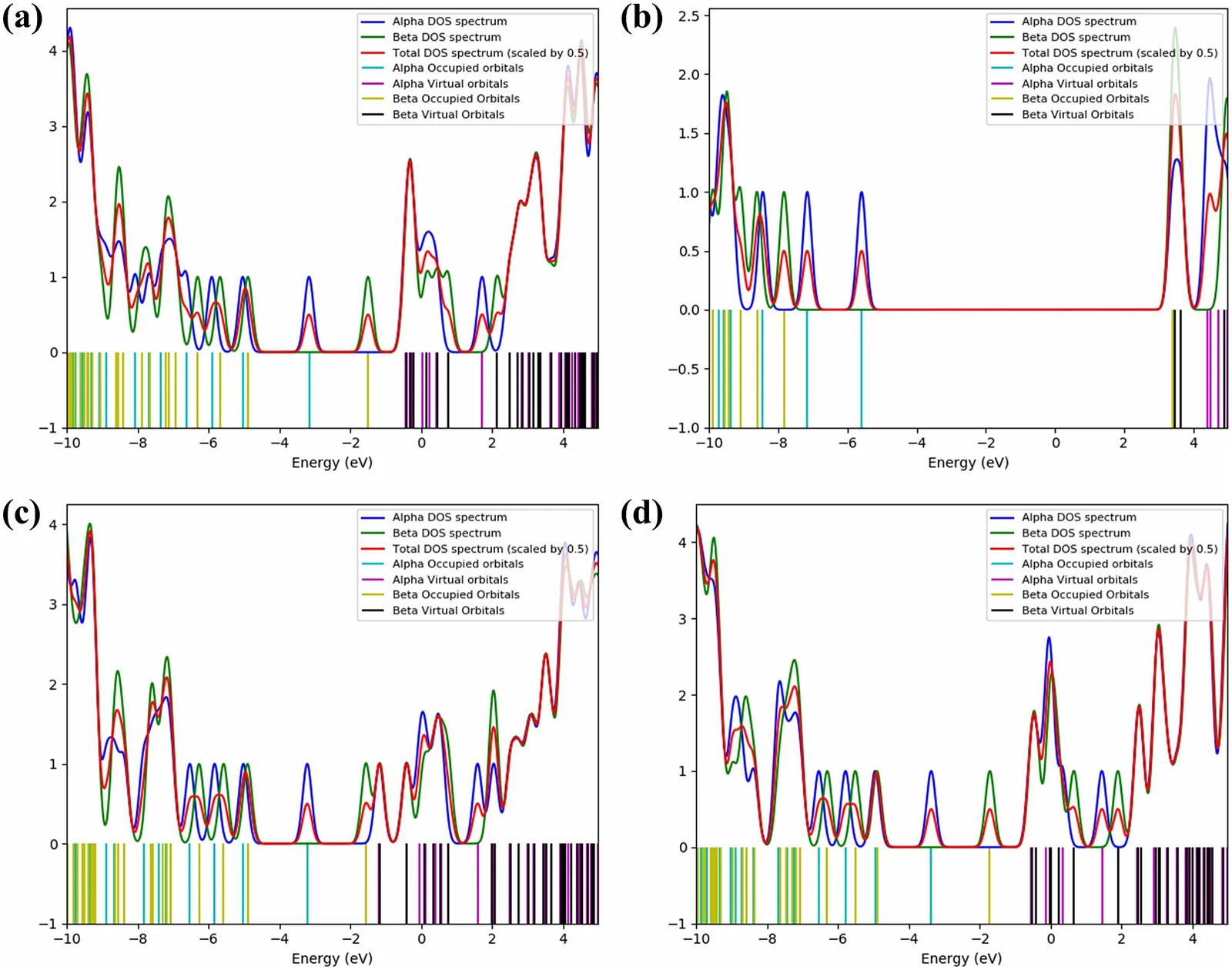
Density of states analysis of post-dock Rhodamine B ligands extracted from protein-ligand complexes. (a) BCL2, (b) BCL2L1, (c) MMP2, and (d) MMP9.

Frontier molecular orbital analysis using density functional theory calculations provides insights into the electronic perturbations and molecular reactivity changes of rhodamine B upon binding to core targets. Density functional theory calculations were performed on both native and post-docking conformations extracted from protein-ligand complexes. The frontier molecular orbital analysis revealed substantial alterations in electronic properties upon target binding (Table 3). Native rhodamine B exhibited HOMO and LUMO energies of −6.01 eV and − 2.48 eV, respectively, with a HOMO-LUMO gap of 3.53 eV, hardness of 1.77 eV, and electrophilicity of 5.11 eV. Upon binding to different targets, rhodamine B demonstrated distinct electronic modifications: BCL2 binding resulted in a reduced HOMO-LUMO gap of 2.66 eV and electrophilicity of 1.16 eV, while BCL2L1 binding yielded a gap of 2.19 eV and the highest electrophilicity of 9.26 eV. Both MMP2 and MMP9 complexes showed similar electronic patterns with gaps of 2.03 eV and 2.82 eV, and electrophilicities of 2.38 eV and 1.37 eV, respectively. The density of states analysis confirmed that rhodamine B ligands extracted from MMP2 and MMP9 complexes exhibited similar electronic distributions, whereas BCL2 and BCL2L1 complexes displayed highly different patterns (Fig. 6).

**Table 3:** Frontier Molecular Orbitals analysis of pre (native) and post-dock poses of Rhodamine B interaction with targets.

Rhodamine B (RB) Poses	HOMO (H) (eV)	LUMO (L) (eV)	Gap (L-H) (eV)	Hardness (ƞ)	Softness (S)	Electro-negativity (χ)	Mean Energy (μ)	Electro-philicity (ω)
RB (Native)	−6.01	−2.48	3.53	1.77	0.28	4.25	−4.25	5.11
RB (BCL2)	−3.09	−0.43	2.66	1.33	0.38	1.76	−1.76	1.16
RB (BCL2L1)	−5.60	−3.41	2.19	1.10	0.46	4.51	−4.51	9.26
RB (MMP2)	−3.22	−1.18	2.03	1.02	0.49	2.20	−2.20	2.38
RB (MMP9)	−3.37	−0.55	2.82	1.41	0.36	1.96	−1.96	1.37

### 3.9 Molecular dynamics simulation

**Fig. 7:**
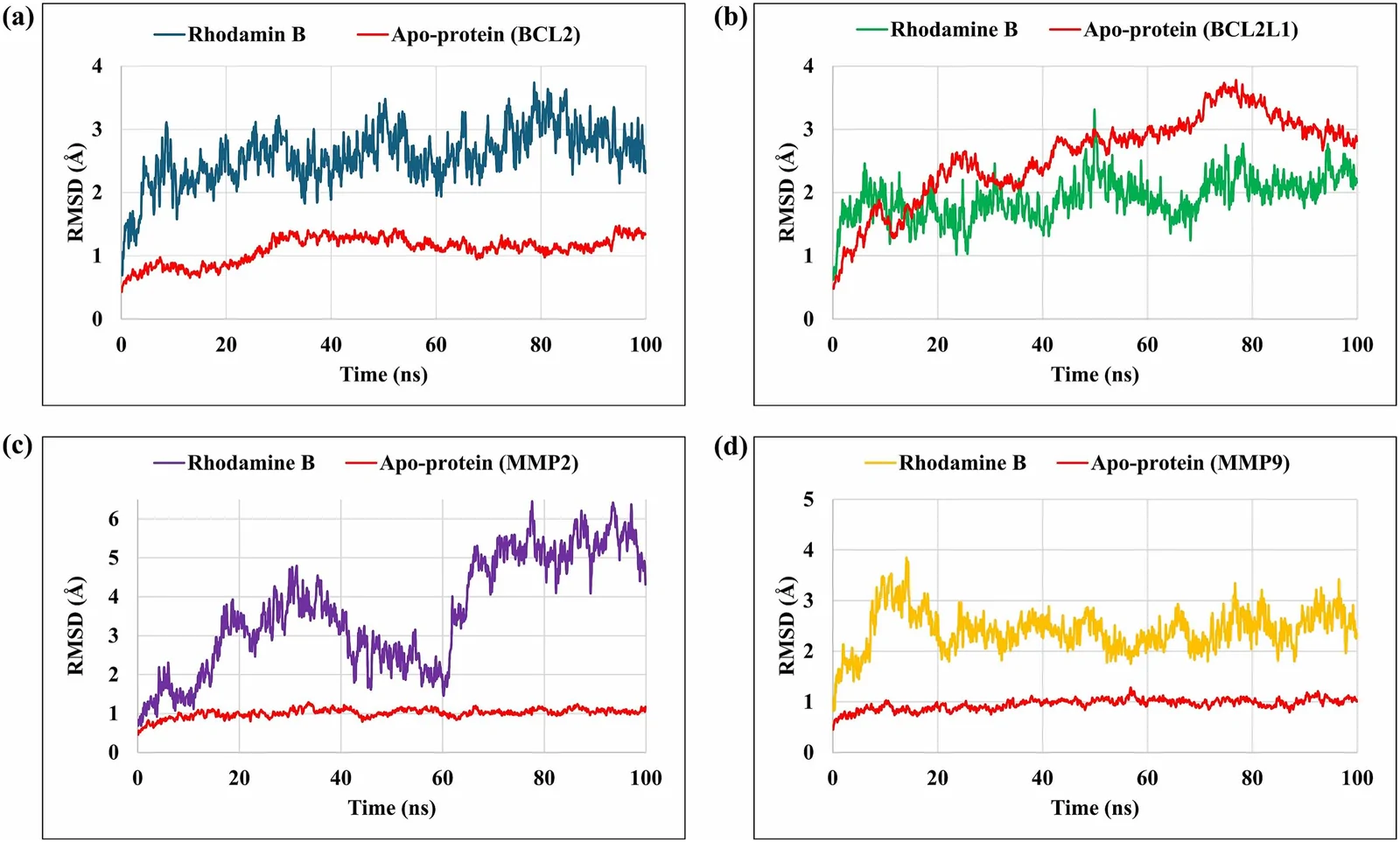
RMSD analysis from 100 ns MD simulation trajectories. Rhodamine B complexes of (a) BCL2, (b) BCL2L1, (c) MMP2, and (d) MMP9.

MD simulations evaluate the conformational stability and binding integrity of Rhodamine B with target apo-proteins over a 100 ns simulation period. The RMSD trajectories revealed distinct stability patterns across the four protein complexes. The Rhodamine B-BCL2 complex exhibited relatively stable behavior with an average RMSD of 2.584 Å for Rhodamine B and 1.345 Å for the apo-protein BCL2, indicating minimal structural fluctuations (Fig. 7a). In contrast, the BCL2L1 complex displayed moderate stability with Rhodamine B maintaining an average RMSD of 1.9124 Å while the apo-protein BCL2L1 showed convergence around 2–3 Å after an initial equilibration phase (Fig. 7b). The MMP2 complex demonstrated the highest conformational variability, with Rhodamine B reaching an average RMSD of 3.595 Å and exhibiting substantial oscillations between 2 and 6 Å throughout the simulation, suggesting potential non-equilibration or dissociation from the binding pocket (Fig. 7c). Conversely, the MMP9 complex showed contrasting behavior, where Rhodamine B maintained an average RMSD of 2.419 Å with relatively consistent fluctuations, while the apo-protein MMP9 remained remarkably stable at approximately 1 Å throughout the entire trajectory (Fig. 7d). These RMSD profiles collectively suggest that Rhodamine B forms stable complexes with BCL2, BCL2L1, and MMP9, whereas the MMP2 complex exhibits greater conformational flexibility. Usually, RMSD values below 3.0 Å indicate that the protein-ligand complex maintains a consistent structural conformation without significant unfolding or ligand displacement over the simulation trajectory.^65^

**Fig. 8:**
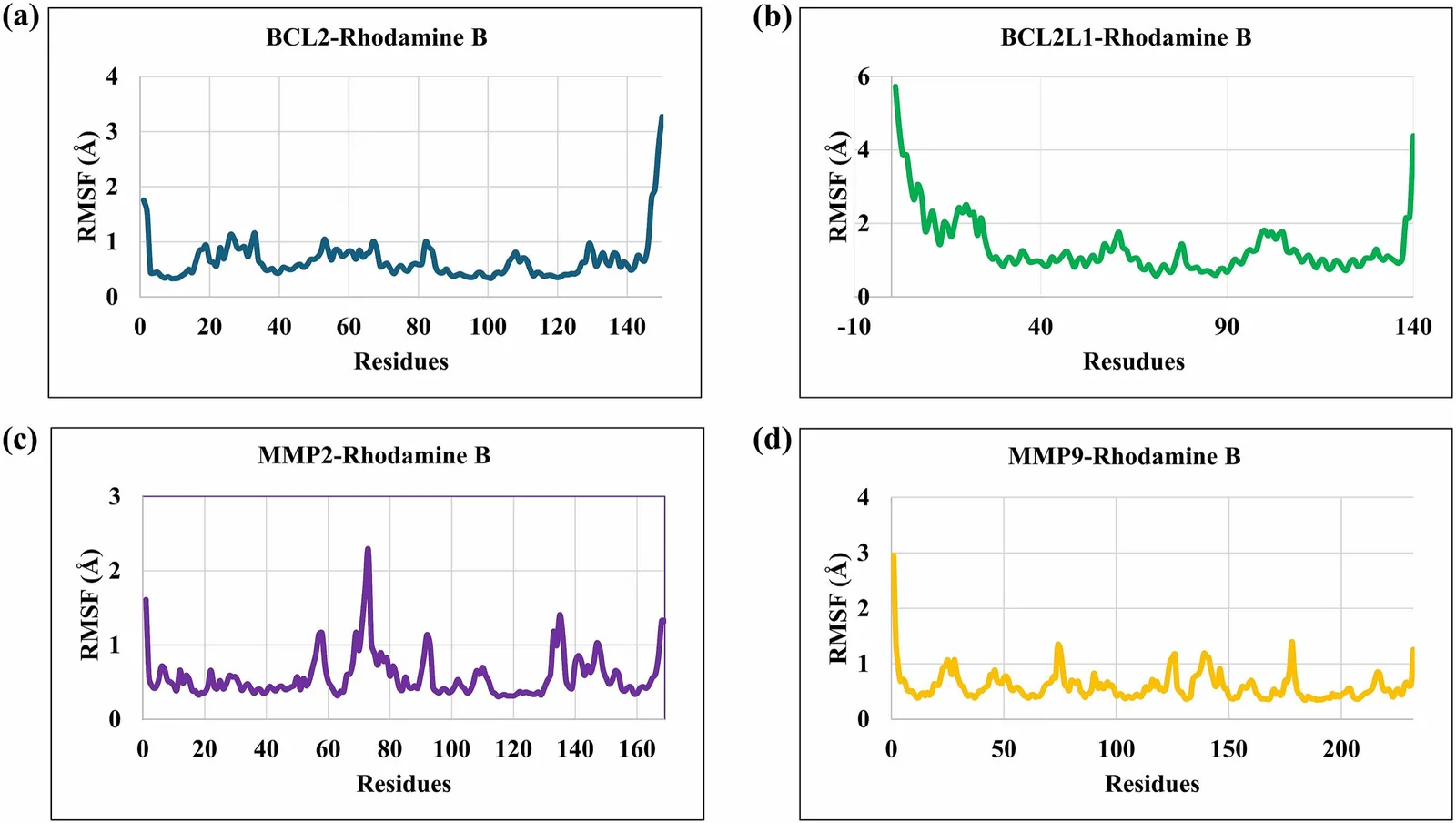
RMSF analysis from 100 ns MD simulation trajectories. Rhodamine B complexes of (a) BCL2, (b) BCL2L1, (c) MMP2, and (d) MMP9.

Additionally, the RMSF analysis revealed distinct flexibility patterns across the four protein-rhodamine B complexes, with BCL2L1 exhibiting the highest average fluctuation (1.349 Å) compared to BCL2 (0.654 Å), MMP9 (0.598 Å), and MMP2 (0.581 Å) (Fig. 8a-d). Notably, MMP2 displayed a prominent flexibility peak at residue 73 with an RMSF value of 2.278 Å, indicating significant local conformational changes in this region during the simulation period (Fig. 8c). Relatively lower average RMSF values were observed for all of the target proteins, BCL2, BCL2L1, MMP2, and MMP9.

**Fig. 9:**
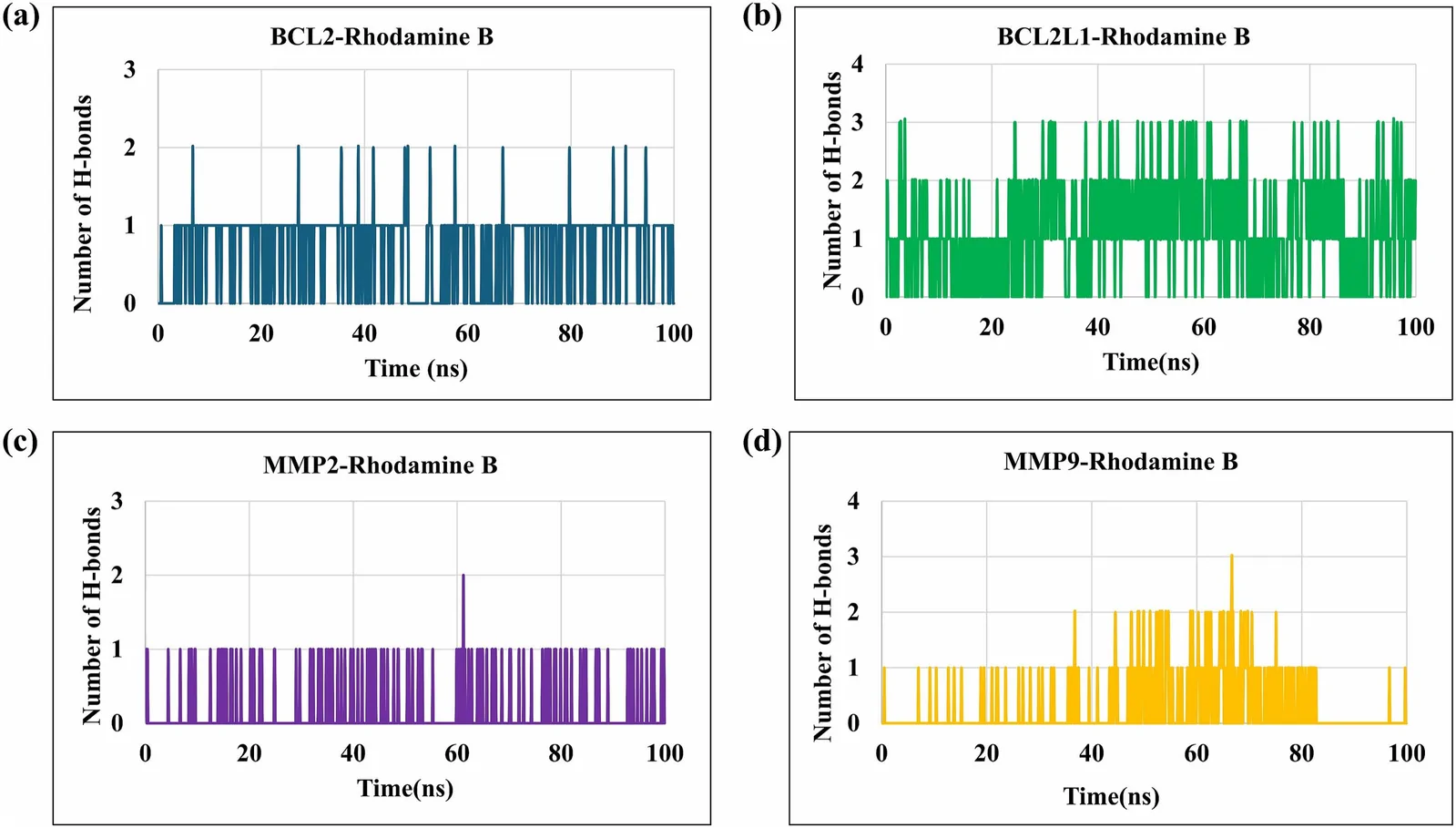
H-bonding analysis from 100 ns MD simulation trajectories. Rhodamine B complexes of (a) BCL2, (b) BCL2L1, (c) MMP2, and (d) MMP9.

Furthermore, hydrogen bonding analysis demonstrated considerable variation in binding stability across the four protein-rhodamine B complexes, with BCL2L1 forming the most extensive hydrogen bond network (average 1.139H-bonds), followed by BCL2 (0.734H-bonds), while MMP9 (0.240H-bonds) and MMP2 (0.137H-bonds) exhibited substantially fewer intermolecular hydrogen bonds. The consistently higher number of hydrogen bonds observed in BCL2 and BCL2L1 complexes suggests stronger electrostatic interactions (Fig. 9**a** and **b**) compared to the matrix metalloproteinase complexes (Fig. 9**c** and **d**). The temporal distribution of hydrogen bonds revealed sustained interactions throughout the simulation period for BCL2 family proteins, whereas MMP2 and MMP9 displayed more transient hydrogen bonding patterns.

### 3.10 Post-dynamics MMPBSA

Post-dynamics MMPBSA calculations quantify the thermodynamic favorability and molecular interactions governing rhodamine B binding to the target proteins to determine the binding free energies and individual energy contributions. The MMPBSA analysis revealed that BCL2 exhibited the most favorable binding affinity with rhodamine B (ΔG total = −32.593 ± 4.721 kcal/mol), followed by MMP9 (−23.439 ± 4.742 kcal/mol), BCL2L1 (−21.909 ± 5.791 kcal/mol), and MMP2 (−11.095 ± 2.709 kcal/mol) (Table 4). The energy decomposition showed that van der Waals interactions were the primary driving force for binding across all complexes, with BCL2 displaying the strongest van der Waals contribution (−60.921 ± 2.815 kcal/mol), while electrostatic interactions varied considerably among the proteins, with MMP9 showing the most favorable electrostatic energy (−21.004 ± 8.092 kcal/mol). The polar solvation energies were unfavorable for all complexes, ranging from 49.397 ± 11.294 kcal/mol for MMP2 to 79.461 ± 6.493 kcal/mol for MMP9, indicating desolvation penalties upon binding, whereas non-polar solvation contributions were consistently favorable, contributing to the overall binding stability through hydrophobic interactions.

**Table 4:** Binding Free Energy Analysis between protein-Rhodamine B complexes using MMPBSA calculations.

ΔG Energy	BCL2-Rhodamine B	BCL2L1-Rhodamine B	MMP2-Rhodamine B	MMP9-Rhodamine B
**ΔG Van der Waals**	−60.921 ± 2.815	−49.867 ± 4.326	−32.921 ± 3.050	−54.919 ± 4.497
**ΔG Electrostatic**	−12.604 ± 7.258	−17.571 ± 6.016	−3.814 ± 7.238	−21.004 ± 8.092
**ΔG Polar Solvation**	68.663 ± 8.245	74.562 ± 7.351	49.397 ± 11.294	79.461 ± 6.493
**ΔG Non-polar Solvation**	−27.731 ± 1.809	−29.035 ± 2.547	−23.757 ± 1.513	−26.979 ± 1.368
**ΔG Gas**	−73.525 ± 9.594	−67.437 ± 9.609	−36.735 ± 9.246	−75.922 ± 6.442
**ΔG Solvation**	40.932 ± 6.028	45.528 ± 5.045	25.640 ± 9.631	52.483 ± 4.810
**ΔG Total**	−32.593 ± 4.721	−21.909 ± 5.791	−11.095 ± 2.709	−23.439 ± 4.742

### 3.11 Free energy landscape and dynamics cross correlation matrix

**Fig. 10:**
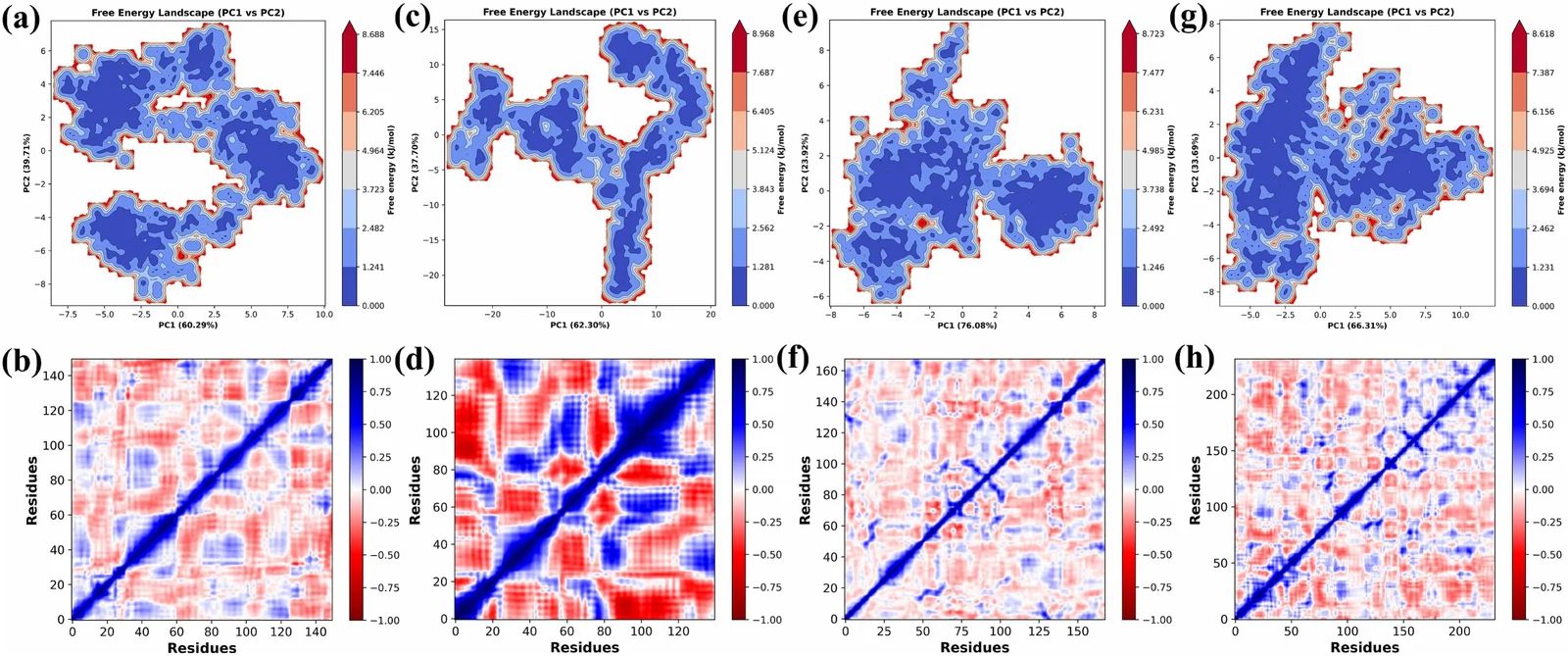
FEL and DCCM. FEL of (a) BCL2-Rhodamine B, (c) BCL2L1-Rhodamine B, (e) MMP2-Rhodamine B, (f) MMP9-Rhodamine B. DCCM of (b) BCL2-Rhodamine B, (d) BCL2-Rhodamine B, (f) BCL2-Rhodamine B, (h) BCL2-Rhodamine B.

Performing free energy landscape (FEL) and dynamic cross-correlation matrix (DCCM) analyses provides insights into understanding the conformational stability and internal motions of protein-ligand complexes during MD simulations. The FEL analysis revealed distinct energy minima patterns for each complex, with BCL2-Rhodamine B (Fig. 10a) displaying multiple stable conformational states distributed across PC1 (60.29%) and PC2 (39.71) coordinates. BCL2L1-Rhodamine B (Fig. 10c) exhibited a more compact energy landscape with primary minima concentrated within PC1 (62.30%) and PC2 (37.70). The MMP2-Rhodamine B complex (Fig. 10e) demonstrated a broader conformational space with PC1 (76.08%) and PC2 (23.92%), while MMP9-Rhodamine B (Fig. 10g) showed the most extensive sampling across PC1 (66.31%) and PC2 (33.69%). The corresponding DCCM analyses (Fig. 10b, d, f, h) indicated that all complexes maintained strong positive correlations along the diagonal regions (correlation coefficients approaching 1.00), with BCL2-related complexes showing more structured correlation patterns compared to BCL2L1, which showed both strongly correlated (dark blue) and anticorrelated (dark red) motions. On the other hand, MMP complexes displayed more diffuse cross-correlations extending beyond 0.50 correlation coefficient thresholds, suggesting greater flexibility and inter-domain communication in the matrix metalloproteinase systems.

### 3.12 Machine learning-based cytotoxicity prediction

**Fig. 11:**
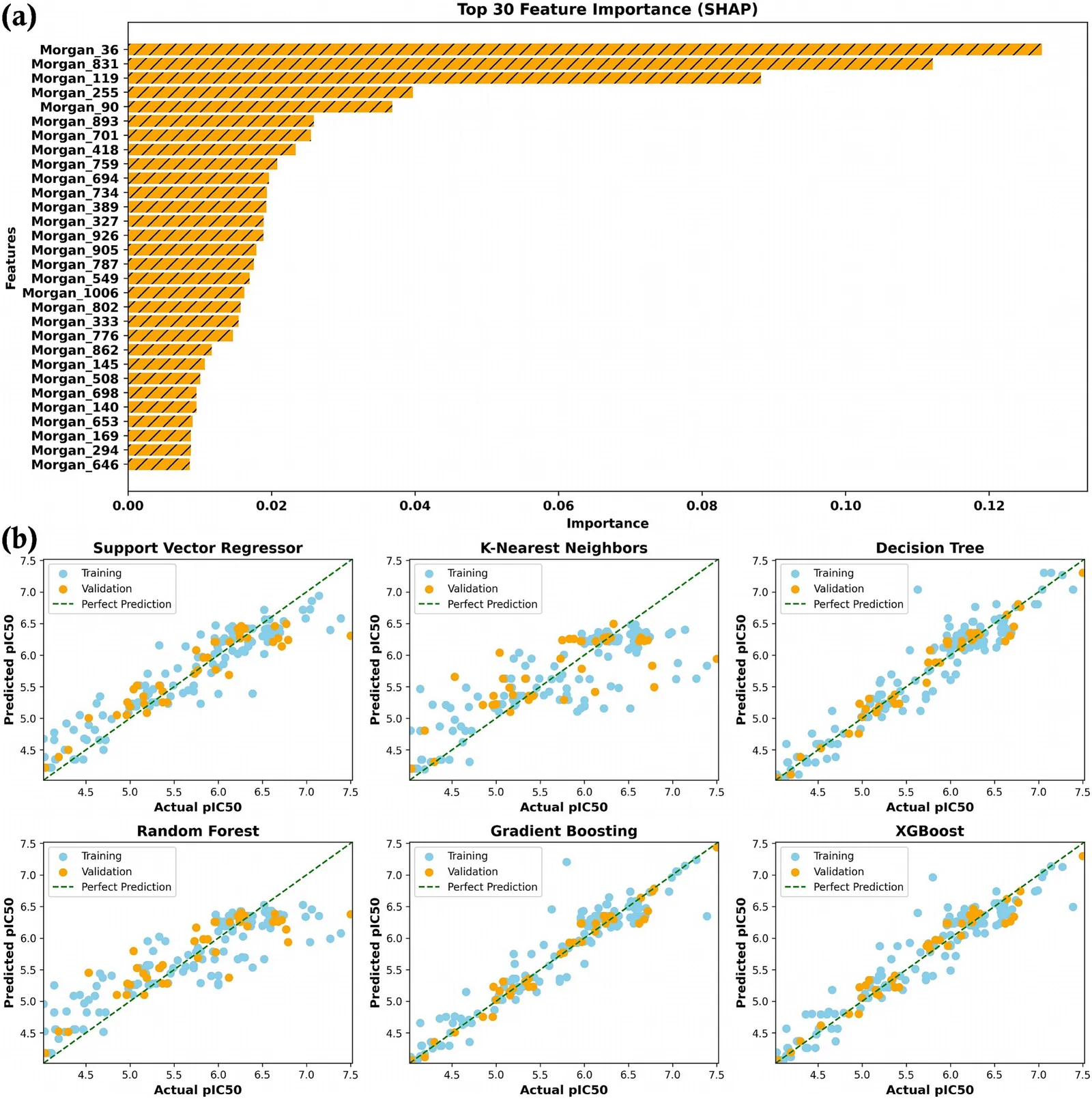
Model development and performance evaluation. (a) Selection of the top 30 most important features based on the SHAP method. (b) Predicted *versus* actual pIC50 scatter plots for six machine learning models: Support Vector Regressor, K-Nearest Neighbors, Decision Tree, Random Forest, Gradient Boosting, and XGBoost, evaluated for cytotoxicity prediction of Rhodamine B in the Caov-3 ovarian cancer cell line. Blue and orange circles denote training and validation sets, respectively, and the dashed line represents perfect prediction. (For interpretation of the references to color in this figure legend, the reader is referred to the web version of this article.)

To evaluate whether the biological activity of Rhodamine B falls within the active (pIC_50_ > 5) or inactive (pIC_50_ < 5) threshold of cytotoxicity, the predictive capability of machine learning models was estimated against the Caov-3 ovarian cancer cell line, which was trained and validated using pIC_50_ (the negative logarithm (base 10) of the half-maximal inhibitory concentration) values as the target variable (Table 5). The dataset used to train the models is presented in **Table S4**. The SHAP-selected Morgan features were presented in Fig. 11a. Among all models, Gradient Boosting demonstrated the most balanced performance, achieving a training R^2^ of 0.862 with a Train MAE of 0.123 and Train RMSE of 0.165, while maintaining a validation R^2^ of 0.779 with a Val MAE of 0.262 and Val RMSE of 0.370, and a cross-validated R^2^ of 0.726 ± 0.066. XGBoost exhibited the second-highest fidelity (Train R^2^ = 0.850; Train MAE = 0.148; Train RMSE = 0.189); with validation performance (Val R^2^ = 0.751; CV R^2^ = 0.732 ± 0.030). The Support Vector Regressor yielded a CV R^2^ of 0.713 ± 0.056, with Val MAE of 0.322 and Val RMSE of 0.472, suggesting consistent generalizability. The Decision Tree model attained a Train R^2^ of 0.925 but showed reduced validation robustness (Val R^2^ = 0.566; CV R^2^ = 0.653 ± 0.076), indicative of overfitting. Random Forest and K-Nearest Neighbors performed comparatively poorly, recording the lowest CV R^2^ values of 0.642 ± 0.050 and 0.557 ± 0.065, respectively, along with the highest cross-validated errors (CV RMSE = 0.494 and 0.548). The predicted *versus* actual pIC_50_ scatter plots confirm these trends, with Gradient Boosting and XGBoost validation points clustering most tightly around the perfect prediction line, while K-Nearest Neighbors exhibited the greatest dispersion (Fig. 11b). Based on the top 3 most reliable models: XGBoost, Gradient Boosting, and Support Vector Regressor, the predicted pIC_50_ of Rhodamine B in Caov-3 ovarian cancer cell line were 4.787, 4.635, 4.139, respectively, fall within the inactive range of cytotoxicity level (pIC_50_ < 5) (Table 6).

**Table 5:** Performance metrics for training, validation, and cross-validation (CV) dataset, including mean absolute error (MAE), root mean square error (RMSE), and coefficient of determination (R^2^) with corresponding standard deviations for six machine learning regression models trained to predict the pIC_50_ values of Rhodamine B in the Caov-3 ovarian cancer cell line.

Models	XGBoost (XGB)	Gradient Boosting (GB)	Support Vector Regressor (SVR)	Decision Tree (DT)	Random Forest (RF)	K-Nearest Neighbors (KNN)
Train MAE	0.148	0.123	0.227	0.156	0.286	0.332
Train RMSE	0.189	0.165	0.307	0.232	0.393	0.475
Train R^2^	0.850	0.862	0.868	0.925	0.783	0.682
Val MAE	0.269	0.262	0.322	0.316	0.360	0.394
Val RMSE	0.393	0.370	0.472	0.519	0.492	0.565
Val R^2^	0.751	0.779	0.641	0.566	0.611	0.485
CV MAE	0.294	0.285	0.318	0.308	0.366	0.386
CV MAE SD	0.036	0.023	0.037	0.029	0.062	0.063
CV RMSE	0.425	0.425	0.440	0.483	0.494	0.548
CV RMSE SD	0.048	0.050	0.067	0.074	0.073	0.075
CV R^2^	0.732	0.726	0.713	0.653	0.642	0.557
CV R^2^ SD	0.030	0.066	0.056	0.076	0.050	0.065

**Table 6:** Predicted output based on machine learning models.

Compound (CID)	SVR	K-NN	DT	RF	GB	XGB
Rhodamine B (6694)	4.139	4.560	4.390	4.710	4.635	4.787

## 4 Discussion

The use of Rhodamine B dye across industrial, textile, food, and laboratory sectors poses a substantial health concern due to its toxicological effects, such as cytotoxicity, genotoxicity, reproductive toxicity, and hepatotoxicity.^13^, ^15^, ^16^ Hence, regulatory authorities worldwide have implemented stringent prohibitions on its use as a food additive and colorant due to the serious health risks associated with human exposure to this compound.^19^

Despite this well-established toxicity profile, the precise molecular mechanisms through which Rhodamine B exerts its reproductive toxic effects in ovarian cancer at the proteomic level remain incompletely understood. Moreover, the molecular landscape of ovarian cancer is dominated by dysregulated apoptosis, matrix remodeling, and estrogen signaling pathways that may be particularly susceptible to the toxic mechanism of Rhodamine B.^4^, ^7^

Therefore, a systematic, network-level understanding of how Rhodamine B's interactions converge upon ovarian cancer-associated molecular targets is of considerable scientific interest, both for understanding the compound's biological hazard profile and for characterizing the mechanistic basis of its cellular toxicity in a cancer-relevant context. Therefore, the present study employed an integrative network toxicology, machine learning, molecular docking, and molecular dynamics simulation framework to address this question.

Systematic multi-database target prediction identified 331 unique molecular targets of Rhodamine B in *Homo sapiens*, reflecting the broad, non-selective binding behavior that is a hallmark of toxic xenobiotic compounds. This promiscuous interaction profile is consistent with Rhodamine B's known capacity to perturb multiple cellular systems simultaneously, a property that underlies its toxicological potency rather than any pharmacological selectivity.^13^, ^15^, ^16^ Cross-referencing these targets against 1164 curated ovarian cancer-associated genes and 1312 differentially expressed genes derived from the GEO datasets GSE4122 and GSE203024 of ovarian cancer revealed 26 shared targets common to all three datasets. This intersection included proteins critically involved in apoptosis regulation (BCL2, BCL2L1), extracellular matrix degradation (MMP2, MMP9), cell cycle progression (TOP2A, CDC25C, AURKA), and hormone signaling (AR, RARA, PPARG), indicating that Rhodamine B's toxic interactions are not incidental to ovarian cancer biology but are mechanistically aligned with the molecular vulnerabilities of ovarian cancer cells.

In addition, network topology analysis identified MMP9, MMP2, BCL2, BCL2L1, AR, KIT, and PPARG as hub targets based on centrality parameters, with MMP9, MMP2, BCL2, and BCL2L1 designated as core targets due to their convergence across multiple ovarian cancer-relevant pathways. Previous studies have demonstrated significant associations between ovarian cancer progression and the expression of anti-apoptotic proteins BCL-2 and BCL2L1, which promote tumor cell survival by inhibiting programmed cell death pathways.^66^, ^67^, ^68^ Additionally, matrix metalloproteinases MMP-2 and MMP-9 have been implicated in ovarian cancer metastasis through their role in extracellular matrix degradation and basement membrane remodeling, facilitating tumor invasion and angiogenesis.^69^, ^70^ Apart from these, existing studies on Rhodamine B have been largely limited to its interaction profiles in model organisms, its binding affinity for human serum albumin, and fragmented pathway-based analyses.^12^, ^35^, ^36^ As a result, the broader, system-level architecture underlying its toxic interactions has yet to be systematically elucidated.

The functional enrichment analysis further revealed that these shared targets are significantly enriched in biological processes associated with collagen catabolism, epithelial cell apoptosis, and cellular response to chemical stress processes that are directly implicated in the cytotoxic disruption of tumor cell homeostasis. Pathway-level enrichment in estrogen signaling, transcriptional misregulation in cancer, and matrix metalloproteinase activation further indicates that Rhodamine B's toxic molecular footprint extensively overlaps with the signaling architecture that sustains ovarian cancer cell survival and invasion. These findings are significant because they suggest that the cytotoxic effects of Rhodamine B in ovarian cancer cells may not be the result of a generalized, non-specific cellular incident, but rather emerge from Rhodamine B's disruptive interaction with a defined set of molecularly critical nodes that govern ovarian cancer cell viability.

In addition, the frontier molecular orbitals analysis demonstrated that Rhodamine B undergoes a HOMO-LUMO gap in the range of 2.03 to 3.53 against all the targets, indicating that Rhodamine B exhibits higher chemical reactivity with the targeted proteins. Furthermore, Molecular docking analysis confirmed that Rhodamine B forms energetically favorable interactions with all four core targets, with binding scores of −7.8 kcal/mol for MMP2, −6.8 kcal/mol for MMP9, −6.7 kcal/mol for BCL2, and − 6.4 kcal/mol for BCL2L1. These binding affinities suggest that Rhodamine B has the potential to interact with the functional binding pockets of these proteins, indicating a plausible mechanism by which it could interfere with their native conformations under sufficient exposure conditions. Moreover, the interaction analysis revealed that Rhodamine B exploits a combination of hydrogen bonding and hydrophobic contacts within each binding pocket, potentially facilitating the extensive steric disruption of catalytic site architecture.

Subsequently, the 100 ns molecular dynamics simulations established that Rhodamine B maintains stable engagement with BCL2, BCL2L1, and MMP9 throughout the simulation period, with average RMSD values of 2.584 Å, 1.912 Å, and 2.419 Å, respectively, while the MMP2 complex exhibited a little bit higher conformational variability (3.595 Å), indicating a more dynamic interaction. Post-dynamics MMPBSA calculations quantified the thermodynamic stability of these toxic interactions, with BCL2 yielding the most favorable binding free energy (ΔG = −32.593 ± 4.721 kcal/mol), followed by MMP9 (−23.439 ± 4.742 kcal/mol), BCL2L1 (−21.909 ± 5.791 kcal/mol), and MMP2 (−11.095 ± 2.709 kcal/mol). The dominance of van der Waals contributions across all complexes, particularly for BCL2 (−60.921 kcal/mol), indicates that Rhodamine B's binding is primarily driven by hydrophobic burial within protein cavities, a mechanism consistent with the compound's amphiphilic chemical structure and its capacity for non-covalent macromolecular disruption. Rhodamine B forms a comparatively weak, metastable interaction with MMP2, unlike the more committed, thermodynamically stable engagement observed for BCL2, BCL2L1, and MMP9. However, MMPBSA is an approximate end-point method that calculates binding free energies qualitatively rather than as absolute thermodynamic values. The sustained hydrogen bonding patterns observed for BCL2 and BCL2L1 complexes throughout the simulation trajectories further support the stability of Rhodamine B's occupancy at critical regulatory sites. Although hydrogen bonding contributed to binding specificity, hydrophobic contacts predominantly drove the overall binding stability across the complexes.

Moreover, the developed top 3 models: XGBoost, Gradient Boosting, and Support Vector Regressor, exhibited a CV R^2^ > 0.70, helping generate an initial hypothesis and indicating the reliability of the models in prediction. The performance aligned with previous models predicting the pIC_50._^51^, ^60^ Moreover, the mean predicted pIC_50_ of Rhodamine B in the Caov-3 ovarian cancer cell line was 4.520 (IC_50_ = 30,199.517 M), which completely falls within the inactive range (pIC_50_ < 5; IC_50_ > 10,000 nM), indicating that Rhodamine B is nowhere close to inhibiting the Caov-3 ovarian cancer cells based on the top 3 models. Despite the favorable docking and MMPBSA scores for BCL2, BCL2L1, and MMP9 indicating strong target engagement, the ML model predicted Rhodamine B to be inactive against Caov-3, suggesting that binding affinity does not necessarily equate to functional inhibition. Whether such engagement results in activation, inhibition, or mere target occupancy depends on factors beyond structural analysis, including conformational activation, cellular uptake, metabolism, and exposure duration. The predicted inactivity therefore does not imply an inability to bind these targets, but rather that binding alone was insufficient to translate into predicted cytotoxicity.

Therefore, based on these computational findings, Rhodamine B is not only unable to inhibit the Caov-3 ovarian cancer cell line, but also maintains stable interactions with core dysregulated targets of ovarian cancer: BCL2, BCL2L1, and MMP9. This suggests that its target binding does not translate into anticancer activity and may instead contribute to undesirable biological effects, indicating potential toxicity.

However, several limitations exist in this study. Firstly, toxicity cannot be definitively concluded from cytotoxicity prediction and binding interactions alone and hence requires additional experiments, such as effects on cell viability, oxidative stress, apoptosis, genotoxicity, or normal-cell damage. Secondly, target predictions are based solely on computational findings and therefore require experimental validation studies. Additionally, sensitivity analysis of targets from different databases to compare how much each database's cutoff affects the final results would add even more confidence. Moreover, docking scores represent an initial rigid-receptor estimate, while our subsequent 100 ns MD simulations were specifically intended to partially compensate by allowing ligand-induced relaxation. For exhaustive thermodynamic characterization, replicates of MD simulation runs are necessary. A further limitation is the relatively small dataset size (201 compounds) used for model development, which may constrain generalizability and hence predictions for structurally dissimilar compounds like Rhodamine B may raise uncertainty. Hence, future studies should prioritize independent external validation to strengthen our findings. Notwithstanding these limitations, this study represents a network-level computational characterization of Rhodamine B's toxic molecular interactions within the dysregulated ovarian cancer targets, predicting BCL2, BCL2L1, and MMP9 as binding-favorable and more dynamically stable engagement targets. Thereby, this furnished hypothesis-driven framework could substantively advance mechanistic toxicological understanding of Rhodamine B in ovarian cancer, providing a structured foundation for prioritizing further experimental studies.

## 5 Conclusion

This study systematically characterized the molecular interactions of Rhodamine B, a well-documented industrial cytotoxic and genotoxic xanthene dye, against ovarian cancer using an integrative network toxicology framework combined with molecular docking, DFT, machine learning, and molecular dynamics simulations, identifying BCL2, BCL2L1, and MMP9 as core convergent targets. The binding favorability of Rhodamine B with BCL2, BCL2L1, and MMP9, along with sustained simulation stability and favorable binding free energies, suggests that Rhodamine B may interact with apoptotic regulatory proteins and matrix metalloproteinases involved in ovarian cancer-associated pathways. Therefore, this study contributes a network toxicology framework for mapping Rhodamine B's molecular interaction landscape onto ovarian cancer-associated targets. Although entirely computational, this study provides a quantitative, target-resolved mechanistic hypothesis for the toxicological relevance of Rhodamine B in ovarian cancer, advancing mechanistic toxicology of synthetic xanthene dyes in gynecological cancer biology and guiding experimental prioritization. Collectively, these findings provide a computational foundation for understanding potential toxic mechanisms of Rhodamine B in ovarian cancer and establish a set of experimentally testable hypotheses for future *in vitro* and *in vivo* validation.

## Declaration of using generative AI in the manuscript preparation process

During the preparation of this work, we used Claude 4.6 Sonnet to improve the language in some cases. After using Claude 4.6, we reviewed and edited the content as needed and took full responsibility for the content of the published article.

## CRediT authorship contribution statement

**Susen Sarker:** Writing – original draft, Visualization, Validation, Methodology, Formal analysis, Data curation, Conceptualization. **Sheikh Abdullah Al Ashik:** Writing – review & editing, Validation, Software, Methodology, Investigation, Formal analysis, Conceptualization.

## Ethics approval and consent to participate

Not applicable.

## Funding

This research received no specific grant from funding agencies in the public, commercial, or not-for-profit sectors.

## Declaration of competing interest

The authors declare that they have no known competing financial interests or personal relationships that could have appeared to influence the work reported in this paper.

## Data Availability

All data generated or analyzed during this study are included in this paper and the supplementary file.

## References

[1] Nersesian S., Glazebrook H., Toulany J., Grantham S.R., Boudreau J.E. (2019). Naturally killing the silent killer: NK cell-based immunotherapy for ovarian cancer. Front Immunol.

[2] Zhang X., Liu Y., Guo B., Li B., Liu H., Wang Z. (2024). Identification of key mRNAs and signaling pathways in obsessive compulsive disorder based on weighted gene co-expression network analysis and cytoHubba plugin. Brain Behav.

[3] Gupta A., O'Cearbhaill R.E., Block M.S. (2024). Vaccination with folate receptor-alpha peptides in patients with ovarian cancer following response to platinum-based therapy: a randomized, multicenter clinical trial. Gynecol Oncol.

[4] Caruso G., Weroha S.J., Cliby W. (2025). Ovarian Cancer: a review. JAMA.

[5] Zheng M., Guo M., Ma F., Li W., Shao Y. (2025). Recent advances in graphitic carbon nitride-based composites for enhanced photocatalytic degradation of rhodamine B: mechanism, properties and environmental applications. Nanoscale Adv.

[6] Zhai H., Huang L., Chen Z. (2017). Chip-based molecularly imprinted monolithic capillary array columns coated GO/SiO2 for selective extraction and sensitive determination of rhodamine B in chili powder. Food Chem.

[7] Amitava K., Khan Dr.A. (2025). A systematic review of the toxicological effects of rhodamine-B. Int J Pharm Sci Rev Res.

[8] Basit F., Van Oppen L.M.P.E., Schöckel L. (2017). Mitochondrial complex I inhibition triggers a mitophagy-dependent ROS increase leading to necroptosis and ferroptosis in melanoma cells. Cell Death Dis.

[9] Macasoi I., Pavel I.Z., Moaca A.E. (2020). Mechanistic investigations of antitumor activity of a rhodamine B-oleanolic acid derivative bioconjugate. Oncol Rep.

[10] Reungpatthanaphong P., Dechsupa S., Meesungnoen J., Loetchutinat C., Mankhetkorn S. (2003). Rhodamine B as a mitochondrial probe for measurement and monitoring of mitochondrial membrane potential in drug-sensitive and -resistant cells. J Biochem Biophys Methods.

[11] Maryanti S.A., Suciati S., Wahyuni E.S., Santoso S., Wayan I., Wiyasa A. (2014). Rhodamine B triggers ovarian toxicity through oxidative stress. Cukurova Medical Journal.

[12] Safitri Y.A., Indrawan I.W.A., Winarsih S. (2015). Rhodamine B induces oxidative stress and cervical epithelial cell proliferation in the uterus. Toxicol Rep.

[13] Liu H.J., He J.R., Guo Y.H. (2026). Green derivative synchronous fluorescence spectroscopy approach for rapid quantification of rhodamine B in food samples. Food Control.

[14] Namlı H., Bişgin A.T. (2024). Simultaneous deep eutectic solvent based microextraction for monitoring brilliant blue and rhodamine B in foodstuff and industrial samples. J Food Compos Anal.

[15] Yusuf T.L., Orimolade B.O., Masekela D., Mamba B., Mabuba N. (2022). The application of photoelectrocatalysis in the degradation of rhodamine B in aqueous solutions: a review. RSC Adv.

[16] Cheng Y.Y., Tsai T.H. (2017). Pharmacokinetics and biodistribution of the illegal food colorant rhodamine B in rats. J Agric Food Chem.

[17] zhu Du R., Zhang Y., Bian Y., yu Yang C., song Feng X., wei He Z. (2024). Rhodamine and related substances in food: recent updates on pretreatment and analysis methods. Food Chem.

[18] Asbollah M.A., Sahid M.S.M., Shahrin E.W.E.S. (2022). Dynamics and thermodynamics for competitive adsorptive removal of methylene blue and rhodamine B from binary aqueous solution onto durian rind. Environ Monit Assess.

[19] Ridjal A.T.M., Kasma A.Y., Aminullah, Basri (2022). Study of rhodamine B dyes content in snacks of Karuwisi traditional market Makassar, South Sulawesi, Indonesia. IOP Conf Ser Earth Environ Sci.

[20] Igberase E., Mkhize I.G. (2026). The adsorption of Rhodamine-B (RHB) by modified chitosan beads in synthetic wastewater: an evaluation of RSM, ANN, and ANFIS techniques in predicting model outcomes. Chem Afr.

[21] Mahlambi M.M., Mishra A.K., Mishra S.B. (2011). Comparison of rhodamine B degradation under UV irradiation by two phases of titania nano-photocatalyst. J Therm Anal Calorim.

[22] Rono N., Kibet J.K., Martincigh B.S., Nyamori V.O. (2020). A comparative study between thermal etching and liquid exfoliation of bulk graphitic carbon nitride to nanosheets for the photocatalytic degradation of a model environmental pollutant, Rhodamine B. J Mater Sci Mater Electron.

[23] Ndlovu L., Bopape M., Aa W. Mhike, Onyango M. (2026). Recycled polypropylene/chitosan-fly ash composites for adsorptive removal of rhodamine B from water. Mater Res Express.

[24] Wu Y., Zhao Q., Wang J. (2024). Boosting peroxymonosulfate activation via CoS/MXene nanocomposite for rhodamine B degradation under simulated sunlight irradiation. Chem Asian J.

[25] Wei H., Li X., Chen L., Li S., Liu H., Song T. (2024). Degradation of Rhodamine B via piezoelectric photocatalyst Bi2WO6 responding to visible light and ultrasound: performance and mechanism. Water Air Soil Pollut.

[26] Samriti A., Ojha J., Prakash (2023). Water challenges in South Asian countries: a focused review on emerging nanomaterials and technological processes in wastewater treatment. ACS EST Water.

[27] Wang M., Nie X., Tian L. (2019). Rhodamine B in spices determined by a sensitive UPLC-MS/MS method. Food Additives & Contaminants: Part B.

[28] Hadi S., Saputri S. Ayu (2019).

[29] Kesuma S., Ikayanti R., Mariana T.W.E., Effendi A.D., Jamal F. (2025). Qualitative analysis of rhodamine b in 4 types of food products in Malang city and regency using thin layer chromatography method. Journal of Scientech Research and Development.

[30] Ragab M., Khaled O., Eissa F., Medhat M. (2025). Ultra-fast LC-MS/MS method for the identification and prevalence of rhodamine B in candies commonly consumed by children in Egypt. Int J Environ Anal Chem.

[31] Asghar W., Akhtar A., ur Rahman H.U., Sami A., Khalid N. (2023). Global perspective on food fraud with special emphasis on the prevalence of food fraud practices and policies in Pakistan. world Food Policy.

[32] Islam M.A. (2020). Food Adulteration and Unethical Use of Formalin. http://www.theseus.fi/handle/10024/344492.

[33] Farid H.M.T., Aman S., Ahmad N. (2023). Ce-doped MoO3 photocatalyst as an environmental purifier for removal of noxious rhodamine B organic pollutant in wastewater. Energy Technol.

[34] Sannino A., Savini S. (2020). A fast and simple method for the determination of 12 synthetic dyes in spicy foods by UHPLC-HRMS. ACS Food Science & Technology.

[35] Li B., Sun Y., Lu J., Peng X. (2021). Investigation on the binding interaction of rhodamine B with human serum albumin: effect of metal ions. J Environ Sci Health B.

[36] Priya P.S., Pratiksha Nandhini P., Vaishnavi S. (2024). Rhodamine B, an organic environmental pollutant induces reproductive toxicity in parental and teratogenicity in F1 generation in vivo. Comparative Biochemistry and Physiology Part C: Toxicology & Pharmacology.

[37] Banerjee P., Kemmler E., Dunkel M., Preissner R. (2024). ProTox 3.0: a webserver for the prediction of toxicity of chemicals. Nucleic Acids Res.

[38] Fu L., Shi S., Yi J. (2024). ADMETlab 3.0: an updated comprehensive online ADMET prediction platform enhanced with broader coverage, improved performance, API functionality and decision support. Nucleic Acids Res.

[39] Gfeller D., Grosdidier A., Wirth M., Daina A., Michielin O., Zoete V. (2014). SwissTargetPrediction: a web server for target prediction of bioactive small molecules. Nucleic Acids Res.

[40] Davis A.P., Wiegers T.C., Johnson R.J., Sciaky D., Wiegers J., Mattingly C.J. (2023). Comparative Toxicogenomics database (CTD): update 2023. Nucleic Acids Res.

[41] Desaphy J., Bret G., Rognan D., Kellenberger E. (2015). Sc-PDB: a 3D-database of ligandable binding sites—10 years on. Nucleic Acids Res.

[42] Pérez V., Aybar C., Pavía J.M. (2021).

[43] Lin Y., Hu Z. (2021). Bioinformatics analysis of candidate genes involved in ethanol-induced microtia pathogenesis based on a human genome database: GeneCards. Int J Pediatr Otorhinolaryngol.

[44] Amberger J.S., Bocchini C.A., Schiettecatte F., Scott A.F., Hamosh A. (2015). OMIM.org: online mendelian inheritance in man (OMIM®), an online catalog of human genes and genetic disorders. Nucleic Acids Res.

[45] Clough E., Barrett T., Wilhite S.E. (2024). NCBI GEO: archive for gene expression and epigenomics data sets: 23-year update. Nucleic Acids Res.

[46] Espe S. (2018). Malacards: The human disease database. J Med Libr Assoc.

[47] Dong H., Li M., Chen H. (2023). Using network pharmacological analysis and molecular docking to investigate the mechanism of action of quercetin's suppression of oral cancer. J Cancer Res Clin Oncol.

[48] Islam K.M.T., Shibly A.Z. (2025). Network and pharmacophore guided and BCL2 and HSP90AA1 targeted drug repurposable approaches against rheumatoid arthritis mediated diffuse large B-cell lymphoma. Int J Biol Macromol.

[49] Szklarczyk D., Nastou K., Koutrouli M. (2025). The STRING database in 2025: protein networks with directionality of regulation. Nucleic Acids Res.

[50] Kohl M., Wiese S., Warscheid B. (2011). Cytoscape: software for visualization and analysis of biological networks. Methods Mol Biol.

[51] Islam K.M.T., Mahmud S. (2025). In-silico exploring pathway and mechanism-based therapeutics for allergic rhinitis: network pharmacology, molecular docking, ADMET, quantum chemistry and machine learning based QSAR approaches. Comput Biol Med.

[52] Liu Y., Cao Y. (2024). Methods Mol Biol.

[53] Islam K.M.T., Islam M.M., Habib J. Bin (2025). In silico approaches unveil the mechanism of action of *Eclipta prostrata* against acute myeloid leukemia. Sci Rep.

[54] Ahamed M.S., Al Ashik S.A. (2026). Rapid screening of potent and mechanistically insightful repurposable anticancer drugs targeting EGFR for non-small cell lung cancer: machine learning-aided and structure-guided approach. Mol Divers.

[55] García-Valverde M., Cordero N.A., de la Cal E.S. (2015).

[56] Islam K.M.T., Mahmud S. (2026). SIRT7 as a context-dependent biomarker and therapeutic target: insights from a pan-cancer study. PLoS One.

[57] Samad A., Imam M.A., Alam D., Ahmed A., Islam A., Parveen S. (2026). Urea induced characterization & structural dynamics of the nucleocapsid protein using biophysical and in silico approaches. J Mol Struct.

[58] Islam K.M.T., Khanam R., Roy A. (2025). Transcriptomics analysis unveils the complex interplay between diabetes and hypertension in regulating renal cell carcinoma pathway followed by pancreatic metastasis. Journal of Genetic Engineering and Biotechnology.

[59] Homeyer N., Gohlke H. (2012). Free energy calculations by the molecular mechanics Poisson-Boltzmann surface area method. Mol Inform.

[60] Ahamed M.S., Al Ashik S.A. (2025). Computer-aided unveiling molecular mechanisms of Xylocarpus granatum against colorectal cancer: therapeutic intervention targeting P13K-AKT signaling pathway. Comput Biol Med.

[61] Al Imran A.Q.M., Sarker S., Priya S.N., Hossain M.R., Al Ashik S.A. (2026). Computational exploration of quercetin derivatives from Azadirachta indica leaf as threonine tyrosine kinase inhibitors for potential lung and pancreatic cancer treatment. Comput Biol Chem.

[62] Gaulton A., Bellis L.J., Bento A.P. (2012). ChEMBL: a large-scale bioactivity database for drug discovery. Nucleic Acids Res.

[63] Bento A.P., Hersey A., Félix E. (2020). An open source chemical structure curation pipeline using RDKit. J Cheminform.

[64] Pedregosa F., Varoquaux G., Gramfort A. (2011). Scikit-learn: machine learning in Python. J Mach Learn Res.

[65] Nasution M.A.F., Bekker G.-J., Re S., Mizuguchi K. (2026). Stability-driven pose prediction and ligand design via contact persistence analysis from molecular dynamics simulations. Comput Struct Biotechnol J.

[66] Olbromski P.J., Bogacz A., Bukowska M. (2023). Analysis of the polymorphisms and expression levels of the BCL2, BAX and c-MYC genes in patients with ovarian cancer. Int J Mol Sci.

[67] Wang C., Qiao X., Wang J. (2022). Amelioration of DMH-induced colon cancer by eupafolin through the reprogramming of apoptosis-associated p53/Bcl2/Bax signaling in rats. Eur J Inflamm.

[68] Xu J., Guo Z., Yuan S., Li H. (2022). BCL2L1 is identified as a target of naringenin in regulating ovarian cancer progression. Mol Cell Biochem.

[69] Ye W., Xue C., Chen L., Chen X., Zhang D. (2025). Silencing of STX4 inhibits the proliferation, migration and invasion of ovarian cancer cells via EMT/MMP2/ CCND1 signaling pathway. J Ovarian Res.

[70] Bandopadhyay S., Dey U., Chakraborty P. (2024). Handbook of Proteases in Cancer: Cellular and Molecular Aspects.

