# Supplementary material for: Network toxicology-aided identification of potential toxicological mechanisms of rhodamine B in ovarian cancer: Insights from transcriptomics, molecular docking, molecular dynamics simulations, and machine learning modeling
Source: J Genet Eng Biotechnol. 2026 Aug 18;24(4):100806. doi: 10.1016/j.jgeb.2026.100806 (PMC13508649; doi:10.1016/j.jgeb.2026.100806)
Supplement: Supplementary file 1 [file mmc1.docx]

**Figure S1.** Validation of the CB-dock2 molecular docking protocol. (A) BCL2 complex, (B) BCL2L1 complex, (C) MMP2 complex, and (D) MMP9 complex. Green = co-crystallized ligand before docking, and red = co-crystallized ligand after docking.
